# Sialylated Autoantigen-Reactive IgG Antibodies Attenuate Disease Development in Autoimmune Mouse Models of Lupus Nephritis and Rheumatoid Arthritis

**DOI:** 10.3389/fimmu.2018.01183

**Published:** 2018-06-06

**Authors:** Yannic C. Bartsch, Johann Rahmöller, Maria M. M. Mertes, Susanne Eiglmeier, Felix K. M. Lorenz, Alexander D. Stoehr, Dominique Braumann, Alexandra K. Lorenz, André Winkler, Gina-Maria Lilienthal, Janina Petry, Juliane Hobusch, Moritz Steinhaus, Constanze Hess, Vivien Holecska, Carolin T. Schoen, Carolin M. Oefner, Alexei Leliavski, Véronique Blanchard, Marc Ehlers

**Affiliations:** ^1^Laboratories of Immunology and Antibody Glycan Analysis, Institute for Nutrition Medicine, University of Lübeck and University Medical Center Schleswig-Holstein, Lübeck, Germany; ^2^Department of Anesthesiology and Intensive Care, University of Lübeck and University Medical Center Schleswig Holstein, Lübeck, Germany; ^3^Laboratory of Tolerance and Autoimmunity, German Rheumatism Research Center, An Institute of the Leibniz Association, Berlin, Germany; ^4^Laboratory of Glycodesign and Glycoanalytics, Institute for Laboratory Medicine, Clinical Chemistry and Pathobiochemistry, Charité – University Medicine Berlin, Berlin, Germany; ^5^Airway Research Center North (ARCN), University of Lübeck, German Center for Lung Research (DZL), Lübeck, Germany

**Keywords:** autoimmunity, IgG glycosylation, sialylation, ST6gal1, systemic lupus erythematosus, rheumatoid arthritis, immunosuppression, Th17

## Abstract

Pro- and anti-inflammatory effector functions of IgG antibodies (Abs) depend on their subclass and Fc glycosylation pattern. Accumulation of non-galactosylated (agalactosylated; G0) IgG Abs in the serum of rheumatoid arthritis and systemic lupus erythematosus (SLE) patients reflects severity of the diseases. In contrast, sialylated IgG Abs are responsible for anti-inflammatory effects of the intravenous immunoglobulin (pooled human serum IgG from healthy donors), administered in high doses (2 g/kg) to treat autoimmune patients. However, whether low amounts of sialylated autoantigen-reactive IgG Abs can also inhibit autoimmune diseases is hardly investigated. Here, we explore whether sialylated autoantigen-reactive IgG Abs can inhibit autoimmune pathology in different mouse models. We found that sialylated IgG auto-Abs fail to induce inflammation and lupus nephritis in a B cell receptor (BCR) transgenic lupus model, but instead are associated with lower frequencies of pathogenic Th1, Th17 and B cell responses. In accordance, the transfer of small amounts of immune complexes containing sialylated IgG Abs was sufficient to attenuate the development of nephritis. We further showed that administration of sialylated collagen type II (Col II)-specific IgG Abs attenuated the disease symptoms in a model of Col II-induced arthritis and reduced pathogenic Th17 cell and autoantigen-specific IgG Ab responses. We conclude that sialylated autoantigen-specific IgG Abs may represent a promising tool for treating pathogenic T and B cell immune responses in autoimmune diseases.

## Introduction

The ability of IgG antibodies (Abs) to modulate immune responses depends on the Ab subclass and the structure of the N-glycan attached to Asn-297 in the Fc region that affect IgG binding to activating and inhibitory Fcγ receptors (FcγRs) on effector cells (1, 2). The biantennary core of the Fc glycan consists of four N-acetylglucosamines (GlcNAcs) and three mannoses, which can be further modified with fucose, bisecting GlcNAc, galactose and terminal sialic acid residues (Figure S1 in Supplementary Material).

The abundance of non-galactosylated (agalctosylated; G0) serum IgG Abs that lack galactose and terminal sialic acid residues positively correlates with the disease severity in rheumatoid arthritis (RA) and systemic lupus erythematosus (SLE) (3–25), whereas alleviated disease activity in RA patients during pregnancy or after anti-TNF treatment is associated with increased levels of sialylated IgG Ab (6, 17, 20, 26–28). Intriguingly, this correlation is especially prominent when only autoreactive IgG Abs are analyzed (22), suggesting that G0 IgG Abs may exacerbate autoimmune inflammation in an antigen-specific manner. Indeed, agalactosylated, but not sialylated, IgG autoantibodies (autoAbs) are able to induce disease symptoms in passive models of arthritis (9, 24).

With regard to the development of differently Fc glycosylated IgG Abs, it has been shown that immune responses under inflammatory conditions induce plasma cells (PCs) that generate G0 IgG, whereas immune responses under tolerogenic conditions induce more galactosylated and sialylated IgG Abs (29–32).

The anti-inflammatory effects of sialylated IgG Abs have first been reported for the intravenous immunoglobulin (IVIG)—pooled human serum IgG from healthy donors (33–35). The sialylated IVIG fraction attenuates arthritis in mice *via* its binding to the C-type lectin receptor SIGN-R1 (specific ICAM-3 grabbing non-integrin-related 1) on regulatory marginal-zone macrophages (36), and thereby induces an anti-inflammatory environment and upregulates the inhibitory Fcγ receptor FcγRIIB on effector macrophages (37). Moreover, sialylated IVIG is able to inhibit dendritic cell (DC) maturation through an FcγRIIB-independent mechanism (29, 38–40). Together, these data suggest that the sialylated IVIG fraction exerts anti-inflammatory effects on both innate and adaptive immune cells.

Under physiological conditions, IgG Abs mediate their effector functions through the formation of immune complexes (ICs) with an antigen (29–32, 41). Recent reports suggest that sialylation of antigen-specific IgG Abs affects their effector functions and the course of an immune response (29, 30). In the context of autoimmunity, application of small amounts of sialylated IgG autoAbs has reduced joint swelling in the collagen-induced arthritis (CIA) model (24). Furthermore, endogenous sialylation of IgG Abs have attenuated disease development in mouse models of nephritis and arthritis through a pathway similar to IVIG (42). Finally, ICs containing sialylated antigen-specific IgG Abs have inhibited LPS-induced IL-6 production by DCs *in vitro* (29).

To further investigate the protective effect of sialylated autoantigen-specific IgG Abs on the development of autoimmune pathology, here we studied the disease course in lupus nephritis-prone FcγRIIB-deficient (Fcgr2b^−/−^) mice and in 56R^+/−^Fcgr2b^−/−^ mice that express a transgenic self- and polyreactive B cell receptor (BCR) (43–46) and produce T cell-independent sialylated IgG2a and IgG2b autoAbs (47). We further tested how sialylated collagen type II (Col II)-reactive monoclonal murine IgG Abs influence the development of Col II-induced arthritis (CIA), accumulation of Th1 and Th17 cells, and autoAb production. Our results suppose that sialylated IgG autoAbs attenuate the development of pathogenic autoimmune conditions and might affect inflammatory T and B cell responses.

## Materials and Methods

### Mice

C57BL/6 wt mice were purchased from Charles River Laboratories (Bar Harbor, ME, USA). Fcgr2b^−/−^ mice and 56R^+/−^Fcgr2b^−/−^ mice with the transgenic VDJ4 heavy (H) chain knock-in (56R) (43, 44) on the C57BL/6 background have been described previously (45, 46, 48–50). Ovalbumin (OVA)-specific TCR transgenic OT-II^+/+^ mice (B6.Cg-Tg(TcraTcrb)425Cbn/J; stock no. 004194) (51) and Thy1.1^+/+^ mice (B6.PL-Thy1a/CyJ; stock no. 000406) on the C57BL/6 background were purchased from Jackson Laboratories. F1 offsprings of OT-II^+/+^ × Thy1.1^+/+^ breedings were used for cell transfer experiments. Genotypes were determined *via* PCR amplification of tail DNA (46). The mice were bred and maintained in accordance with federal laws and institutional guidelines.

### Reagents

For the experiments, ovalbumin (OVA) was purchased from Sigma-Aldrich (Steinheim, Germany) and 2,4,6-Trinitrophenyl (TNP)(12)-coupled bovine serum albumin (TNP(12)-BSA) and TNP(5)OVA were purchased from Biosearch Technologies (Novato, CA, USA or Petaluma, CA, USA). TNP-sheep IgG was prepared using TNP-e-aminocaproyl-OSu (Biosearch Technologies) and sheep IgG (Sigma-Aldrich) in the laboratory. IVIG (Intratect) was obtained from Biotest Pharma GmbH (Dreieich, Germany). Complete Freund’s adjuvant [CFA; 1 mg Mycobacterium tuberculosis (*Mtb*)/ml; #F5881] and incomplete Freund’s adjuvant (IFA; #F5506) were purchased from Sigma-Aldrich. Enriched CFA (eCFA) was prepared by adding heat-killed *Mtb*.H37 RA (BD Biosciences, San Diego, CA, USA) to IFA (5 mg *Mtb*/ml) (30).

### Detection of Proteinuria

Urine samples were tested on Multistix 10 Visual strips (Bayer, Leverkusen, Germany). Proteinuria was scored as follows: 0 = negative, 1 = ≤ 75 mg/dl, 2 = ≤ 125 mg/dl, 3 = > 125 mg/dl.

### Kidney Histology

Kidney specimens of lupus-prone Fcgr2b^−/−^ or 56R^+/−^Fcgr2b^−/−^ mice were embedded in Tissue-Tek OCT compound immediately after removal and snap frozen on dry ice. Sections (7 µm) were fixed in ice-cold acetone and stained with FITC-conjugated anti-mouse IgG2a_a_ or IgG2a_b_ (Bethyl Laboratories; Montgomery, TX, USA), Cy5-conjugated anti-mouse Mac-1 (M1/70.15.11) and Cy5-conjugated anti-mouse macrophage marker (F4/80).

### HEp-2 Cell Staining

Sera (1:100 dilution) from lupus-prone Fcgr2b^−/−^ or 56R^+/−^Fcgr2b^−/−^ mice were added to commercially available HEp-2 slides (Orgentec, Mainz, Germany). The captured Abs were detected with a FITC-conjugated anti-mouse IgG2a_a_ or IgG2a_b_ Ab (Bethyl Laboratories).

### Flow Cytometric Analysis

Indicated organs from immunized and untreated mice were prepared for flow cytometric analysis (LSRII, BD Biosciences or Attune; Thermo Fisher Scientific, Waltham, MA, USA) on the indicated days. The following biotin- or fluorochrome-coupled Abs were used for staining at 4°C: anti-CD138 (clone 218-2), anti-B220 (RA3-6B2), anti TCRbeta (H5-590), anti-CD95 (Jo-2), anti-CD4 (RM4-5), anti-IgM_a_ (DS-1), anti-IgM_b_ (AF6-78), anti-IL-17A (TC11-18H10), anti-IFNγ (XMG1.2) (all purchased from BD Biosciences), anti-CD8 (53-6.7), anti-GL-7 (GL-7), anti-IgG1 (RMG1-1), anti-CD90.1/Thy1.1 (Ox-7) (all purchased from Biolegend, San Diego, CA, USA), anti-Foxp3 (FJK16s), anti-IgM (eB121-15F9) (all purchased from Thermo Fisher Scientific), anti-IgG (polyclonal; Bethyl Laboratories), anti-St6gal1 (polyclonal; R&D Systems, Minneapolis, MN, USA), anti-CD44 (IM7) and anti-CD62L (MEL14) (all of which were generated in the laboratory). Fluorochrome-coupled OVA was purchased from Thermo Fisher Scientific and streptavidin reagents from Biolegend. For intracellular staining, the samples were fixed with Cytofix/Cytoperm according to the manufacturer’s instructions (BD Biosciences) followed by permeabilization with Perm/Wash Buffer (own preparation, 0.05% saponin in 0.05× PBS). For intranuclear Foxp3 staining, samples were fixed and permeabilized with the Foxp3 Fix/Perm buffer set according to the manufacturer’s instructions (Thermo Fisher Scientific). For intracellular cytokine analysis, cells were re-stimulated with PMA (10 ng/ml) and ionomycin (1 µg/ml) (Sigma-Aldrich) for 4 h, whereby Brefeldin A (Sigma-Aldrich) was added after 1 h of stimulation to facilitate the accumulation of cytokines in the interior of the cell.

### Enzyme-Linked Immunofluorescence Assays (ELISAs)

Abs specific for double-stranded DNA were detected as described previously (46). Briefly, ELISA plates were precoated with 5 µg/ml of methylated BSA (Sigma-Aldrich), followed by overnight incubation at 4°C with 50 µg/ml of calf thymus DNA (Sigma-Aldrich). After washing, the plates were blocked (PBS, 3% BSA, 1 mM EDTA, 0.1% gelatin) and subsequently incubated with 1/100 diluted serum. Captured Abs were detected with horseradish peroxidase-coupled goat anti-mouse IgG, IgG1, IgG2a_a_, IgG2a_b_ or IgG2b secondary Abs (Bethyl Laboratories), followed by incubation with a 3,3′,5,5′-tetramethylbenzidine substrate solution (BD Biosciences); the optical density was measured at 450 nm. Abs against nucleosomes were detected in 1/100 diluted sera using nucleosome-coupled ELISA plates (Orgentec). For the detection of TNP- or Col II-reactive Abs, ELISA plates were coated with 5 µg/ml of TNP-BSA or 2 µg/ml of Col II in 0.05 M Carbonate/Bicarbonate buffer, pH 9.6 (Sigma-Aldrich).

### Depletion of CD4^+^ T Cells

For depletion of CD4^+^ T cells, mice were injected intraperitoneally (i.p.) with 250 µg of anti-mouse CD4 (GK1.5) every 4 days for the indicated period of time. GK1.5 hybridoma Abs were purified from hybridoma cultures using protein G Sepharose. The depletion of CD4^+^ T cells (blood samples) was verified *via* flow cytometry (Figure S2 in Supplementary Material).

### Sialylation Analysis of Serum IgG Abs From wt, Fcgr2b^−/−^ and 56R^+/−^Fcgr2b^−/−^ Mice

Serum IgG Abs from the indicated wt, Fcgr2b^−/−^ and 56R^+/−^Fcgr2b^−/−^ mice were purified using protein G Sepharose. To characterize the sialylation of purified IgG Abs, the GlykoScreen™ Sialic Acid Quantification Kit (Prozyme, Hayward, CA, USA) was utilized according to the manufacturer’s instructions. In brief, sialic acid molecules were enzymatically released from the purified IgG Abs by incubation with sialidase A for 2 h at 37°C. Released sialic acid molecules were enzymatically converted in a two-step process to acetylphosphate and hydrogen peroxide. Addition of HRP catalyzed a reaction of hydrogen peroxide with another added substrate into a fluorescent dye, which was quantified at 590 nm.

### Purification of Polyreactive Serum IgG Abs From 56R^+/−^Fcgr2b^−/−^ Mice

Serum IgG Abs from 56R^+/−^Fcgr2b^−/−^ mice were purified with Protein-G-Sepharose. Purified IgG Abs were applied to TNP(12)-BSA-coupled cyanogen bromide-activated Sepharose 4B (GE Healthcare, Fairfield, CT, USA) columns (prepared in the laboratory) for purification of polyreactive IgG Abs. Reactivity against various autoantigens was verified *via* ELISA (data not shown) and IgG N-glycosylation was characterized through MALDI-TOF mass spectrometry (MS).

### *In Vitro* De-Sialylation of IgG Abs

De-sialylation of purified polyreactive serum IgG Abs was performed with the Prozyme Sialidase kit (Prozyme).

### *In Vitro* Galactosylation and/or Sialylation of IgG Abs

*In vitro* galactosylation and/or sialylation of monoclonal anti-TNP murine IgG1 (clone H5) and anti-Thy1.1 murine IgG1 (clone OX-7) hybridoma Abs (52, 53) and cloned and produced anti-Col II murine IgG1 Abs (see below) were performed as described previously (29, 35). Briefly, Abs were galactosylated with human beta1,4-galactosyltransferase and UDP-Galactose and/or sialylated with human beta-galactoside alpha2,6-sialyltransferase (St6gal1) and CMP-sialic acid (all reagents were obtained from Calbiochem, Darmstadt, Germany). Antigen-reactivity was verified *via* ELISA, and IgG N-glycosylation was analyzed through MALDI-TOF MS or HPLC (32).

### Glycan Analysis of Polyreactive Serum IgG Abs From 56R^+/−^Fcgr2b^−/−^ Mice and Monoclonal IgG Abs *via* MALDI-TOF MS

N-glycans were isolated from purified IgG samples *via* hydrolysis with recombinantly expressed endoglycosidase S (EndoS) from *Streptococcus pyogenes* (54). EndoS cleaves the Fc N-glycans of IgG Abs between the first and second GlcNAc (Figure S1 in Supplementary Material). The resulting N-glycans were purified through solid phase extraction using reversed-phase C18 and graphitized carbon columns (Alltech, Deerfield, IL, USA). The samples were then permethylated according to standard protocols (30, 55) and further investigated *via* MALDI-TOF MS in duplicate. The spectra were recorded on an Ultraflex III mass spectrometer (Bruker Daltonics Corporation, Billerica, MA, USA) equipped with a Smartbeam laser. Calibration was performed on a glucose ladder, and 2,5-dihydroxybenzoic acid was used as the matrix. Spectra were recorded in reflector positive ionization mode, and mass spectra from 3,000 laser shots were accumulated. Based on the terminal sugar moiety, the EndoS resulting peaks were assigned to one of the following nine groups: G0+ bisecting GlcNAc, G0 w/o bisecting GlcNAc, G1+ bisecting GlcNAc, G1 w/o bisecting GlcNAc, G2+ bisecting GlcNAc, G2 w/o bisecting GlcNAc, G1S1, G2S1 and G2S2 (Figure S1 in Supplementary Material). Peaks containing both sialic acid and bisecting GlcNAc were not detected. In general, murine IgG Abs hardly showed bisecting GlcNAc structures. However, the calculated proportions of the bisecting GlcNAc versions of G0, G1 and G2 were added to the percentages of the G0, G1 and G2 versions without bisecting GlcNAc, respectively, to get six groups totaling 100%: G0, G1, G2, G1S1, G2S1 and G2S2. In some figures the percentages of S1 (G1S1 + G2S1) and S2 (G2S2) glycans are presented.

### Nephrotoxic Nephritis-Induced Mouse Model

Nephritis was induced by injection of 100 µg of sheep IgG Abs in CFA on day 0, followed by intravenous (i.v.) injection of 80 µl of sheep anti-glomerular basement membrane (anti-GBM) nephrotoxic serum (NTS) 4 days later (56). Development of nephritis was verified by the detection of proteinuria as described above.

### Cloning and Production of Col II-Reactive Murine IgG1 Abs

The variable VDJ heavy chain and VJ light chain DNA sequences of Col II-reactive murine IgG2b, clone M2139 (VDJ heavy chain: NCBI accession number Z72462; VJ light chain: NCBI accession number Z72463) (57) and IgG2a, clone CII 1-5 (VDJ heavy chain: NCBI accession number MMU69538; VJ light chain: NCBI accession number MMU69539) (58), were synthesized (Mr. Gene, Germany) with flanking restriction sites and cloned into previously described eukaryotic IgH and IgL expression vectors (59, 60), which were modified to include the murine C57BL/6 IgG1 heavy chain or kappa light chain constant region, respectively (Figure S4 in Supplementary Material). The constant heavy and light chain regions were amplified from C57BL/6 splenic cDNA *via* RT-PCR (IgG1 forward primer, 5′-GCGTCGACGACACCCCCATCTGTCTATCCACTGGCCC and reverse primer, 5′-TTATTCGGCGTACGCGTCATTTACCAGGAGAGTGGGAG; kappa forward primer, 5′-GCCGTACGGATGCTGCACCAACTGTATCCAT and reverse primer, 5′-TTATTCGGAAGCTTTCAACACTCATTCCTGTTGAAG). Col II-reactive monoclonal IgG1 Abs were produced *via* polyethylenimine (PEI; Sigma-Aldrich)-mediated cotransfection of human embryonic kidney 293 cells with plasmid DNA encoding the IgH and IgL chains (Figure S4 in Supplementary Material). IgG Ab integrity was analyzed through SDS gel electrophoresis, while Ab reactivity was controlled *via* ELISA, and IgG Fc N-glycosylation was analyzed using MALDI-TOF MS.

### Chicken COL2-Induced Arthritis (CIA) Mouse Model

Chicken type II collagen (Col II; Sigma-Aldrich) was dissolved at 2 mg/ml in 0.05 M acetic acid and emulsified in an equal volume of enriched CFA (see reagents). Then, 8–10-week-old Fcgr2b^−/−^ mice were immunized subcutaneously (s.c.) with 100 µl of the emulsion (equivalent to 100 µg of Col II). On day 21, a booster s.c. injection of 100 µg of chicken Col II in IFA was administered. Mice were monitored for swelling encompassing the paw and ankle or ankylosis of the limb to determine the onset and severity of the disease in a blinded manner. The swelling of each foot was scored as follows: healthy paws and ankles (score 0) showed no abnormal swelling, redness, contact sensitivity or motor activity alterations. Low swelling of paws and/or ankles was scored with 1, pronounced swelling with 2 and severe balloon-like whole swelling (ankylosis) with 3; thus, each mouse could achieve a maximum score of 12. The mean clinical score was calculated by totaling the scores of all mice in a group and dividing by the number of mice in that group. Prevalence indicates the percentage of animals in an individual group with a score >0 on the indicated time point. Onset of disease was specified at the indicated day, at which an animal reached score >0 for the first time.

### Ankle Histology

Ankle samples were embedded in paraffin and sections were stained with hematoxylin and eosin (H&E) or anti-CD3. Immunofluorescence was detected using a Leica DM IRE confocal laser scanning microscope.

### OT-II Cell Transfer Experiments

Purified splenocytes of OT-II^+/−^ × Thy1.1^+/−^ donor mice (8–12-week-old mice) were labeled with the CellTrace Violet Cell Proliferation Kit according to the manufacturer’s protocol (Thermo Fisher Scientific). 3 × 10^7^ labeled cells were transferred i.v. in naive recipient C57BL/6 wt or Fcgr2b^−/−^ mice (8–12-week-old mice). One hour later the recipient mice were injected i.p. with 90 µg of low-sialylated or *in vitro* galactosylated plus sialylated anti-TNP IgG1 (H5) Abs or PBS. On the following day, 200 µl of an 1:1 water-in-oil emulsion of enriched CFA and PBS containing 30 µg of TNP(5)-OVA (Biosearch Technologies) or OVA (Sigma-Aldrich) was injected into each of the recipient mice. After 4 days, the mice were sacrificed and splenic and mesenteric lymphnode cells were analyzed *via* flow cytometry.

### DC Culture

Bone marrow (BM)-derived DCs were generated over 8 days in IMDM (Thermo Fisher Scientific) containing 10% FCS, 10 ng/ml IL-4, 20 ng/ml GM-CSF (R&D Systems) and 50 µM 2-mercaptoethanol. Subsequently, the cells were cultured in 96-well plates with ICs containing 10 µg of chicken Col II and different proportions of non-sialylated (<1% sialylation) and sialylated (46% sialylation) Col II-specific IgG1 (clone M2139; total amount per well: 40 µg/ml) in medium containing 10% IgG-depleted FCS. The IL-6 concentration was detected after 36 h *via* ELISA (BD Biosciences).

### Statistical Analysis

Statistical analyses unless otherwise stated, were performed using Student’s *t*-test comparing two groups or One-way ANOVA for more groups, respectively, or the logrank test for survival curves: *P < 0.05, **P < 0.01, and ***P < 0.001. If not stated otherwise, murine data were taken from one representative out of 2–5 individual experiments or combined from multiple experiments and are presented as the mean or median (median fluorescence intensity) values as indicated ±SEM; each data point represents an individual animal.

## Results

### 56R-Derived IgG2a and IgG2b autoAbs Fail to Induce Disease Symptoms in Lupus-Prone Mice

**Figure 1:**
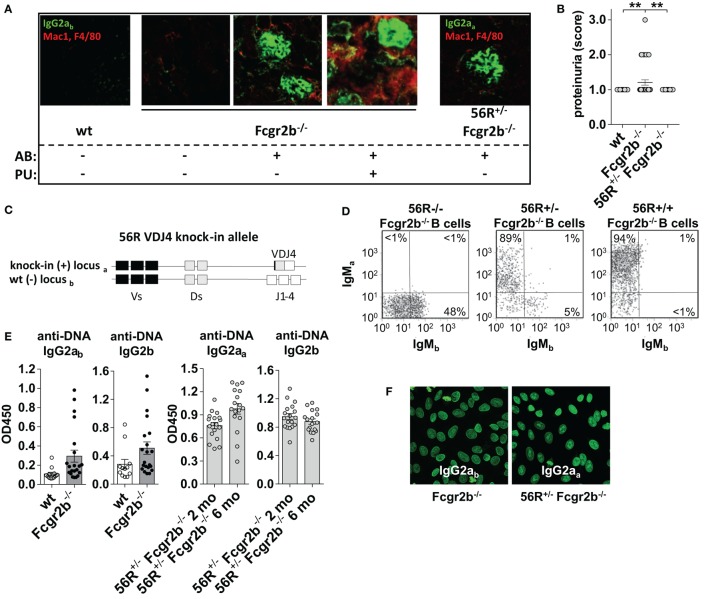
56R^+/−^Fcgr2b^−/−^ mice develop self- and polyreactive IgG2a and IgG2b autoantibodies (autoAbs) earlier than Fcgr2b^−/−^ but show no signs of renal inflammation. **(A)** Indirect immunofluorescence analysis of IgG2a immune complex (IC) depositions and macrophage infiltration in kidney sections from 6-month-old wt mice, IgG autoAb (AB)-negative and proteinuria (PU)-negative (AB^−^PU−), AB^+^PU− and AB^+^PU^+^Fcgr2b^−/−^ mice (all IgG2a_b_) and 56R^+/−^Fcgr2b^−/−^ mice (IgG2a_a_). The presented immunofluorescence images are representative of at least five mice per group. **(B)** PU scores for 5–6-month-old wt (*n* = 30), Fcgr2b^−/−^ (*n* = 39) and 56R^+/−^Fcgr2b^−/−^ (*n* = 30) mice. Each data point represents an individual animal; horizontal lines represent mean values. **(C)** Schematic representation of the self- and polyreactive 56R VDJ4 knock-in (haplotype a) and the wt (haplotype b) heavy chain loci. The 56R VDJ4 knock-in replaced the endogenous IgH Js and can class-switch to all Ab isotypes (43–46). **(D)** The frequencies of B220+ B cells expressing the knock-in allele (a) or the wt allele (b) were analyzed by flow cytometry of splenocytes from 2-month-old 56R^−/−^, 56R^+/−^ or 56R^+/+^Fcgr2b^−/−^ mice. About 5–10% of B cells in 56R^+/−^Fcgr2b^−/−^ mice expressed the wt (IgM_b_+) BCR. **(E)** Anti-DNA IgG2a_b_, IgG2b or IgG2a_a_ subclass Abs in sera of 5–7-month-old wt and Fcgr2b^−/−^ mice and 2- or 6-month-old 56R^+/−^Fcgr2b^−/−^ mice were determined *via* enzyme-linked immunofluorescence assay. Each data point represents an individual animal. Bars indicate mean values. **(F)** IgG2a_b_ and IgG2a_a_ Ab binding to HEp-2 slides from sera of 6-month-old Fcgr2b^−/−^ and 56R^+/−^Fcgr2b^−/−^ mice, respectively.

To study how IgG Fc glycosylation is associated with the development of autoimmune pathology, we first used lupus-prone FcγRIIB knockout mice, a model of spontaneous lupus nephritis (Fcgr2b^−/−^ females on the C57BL/6, haplotype b, background) (46, 48–50). By 4–5 months of age, more than a half of Fcgr2b^−/−^ mice developed DNA-, nucleosome- and poly (here shown for anti-TNP reactivity)-reactive IgG2a and IgG2b autoAbs, which accumulated in the kidney (Figure 1; Figure S2 in Supplementary Material) (46, 49, 50, 60–64).

Fcgr2b^−/−^ mice positive for IgG autoAbs started to develop proteinuria by the age of 6 months, and about a half of the Fcgr2b^−/−^ mice died due to severe nephritis by the age of 9 months (Figures 1A,B and data not shown) (46, 49, 50). Mice developing nephritis showed, in addition to IgG Ab depositions, also macrophage infiltrations in the kidney (Figure 1A). Inversely, mice with IgG Ab depositions in the kidney, but without macrophage infiltrations, did not show proteinuria and nephritis (Figure 1A).

In comparison, we analyzed nephritis development in female Fcgr2b^−/−^ mice that expressed one “knock-in” allele of the self- and polyreactive 56R VDJ4 Ig heavy chain (haplotype a) (56R^+/−^Fcgr2b^−/−^ mice; Figure 1) (43–46). About 90–95% of all B cells in 56R^+/−^Fcgr2b^−/−^ mice expressed a 56R-based BCR (Figure 1D).

Earlier studies have shown that 56R^+/−^ mice on the C57BL/6 wt background produce autoreactive IgM and some IgG Abs (47), and that the introduction of the 56R allele into lupus-prone Fcgr2b^−/−^ mice lead to increased IgG class switched autoAbs, particular of the 56R allele (45, 46). Whereas the generation of IgG2a and IgG2b autoAbs in Fcgr2b^−/−^ mice requires T cell help (50, 60), autoAbs in 56R^+/−^ wt mice largely develop in a T-cell-independent manner (47). Because we recently demonstrated that T cell-independent B cell activation induces immunosuppressive sialylated IgG Abs *in vivo* (30), we wondered whether the introduction of the 56R allele into lupus-prone Fcgr2b^−/−^ mice may lead to T cell-independent IgG autoAbs and provide a disease-protective effect.

In line with previous reports, 56R^+/−^Fcgr2b^−/−^ mice generated high serum titers of class-switch DNA-, nucleosome- and polyreactive IgG2a_a_ and IgG2b Abs, which formed depositions in the kidney (Figure 1; Figure S2 in Supplementary Material) (45, 46). In contrast to Fcgr2b^−/−^ mice, all 56R^+/−^Fcgr2b^−/−^ mice developed IgG2a_a_ and IgG2b autoAbs already by the age of 2 months (45, 46), which (IgG2a_a_, but not IgG2b) only slightly further increased until the age of 6 months (Figure 1E). We also found comparable anti-nuclear reactivity of IgG2a Abs in the sera of 5–7 months old Fcgr2b^−/−^ and 56R^+/−^Fcgr2b^−/−^ mice (Figure 1F). However, despite the early presence of IgG Abs of similar antigen specificity and subclass, and IgG Ab deposition in the kidney, none of the 56R^+/−^Fcgr2b^−/−^ mice showed macrophage infiltration into the kidney and proteinuria by the age of 9 months (Figures 1A,B).

### 56R^+/−^Fcgr2b^−/−^ Mice Do Not Develop Splenomegaly and Do Not Accumulate Th1 and Th17 cells and PCs Seen in Fcgr2b^−/−^ Mice

**Figure 2:**
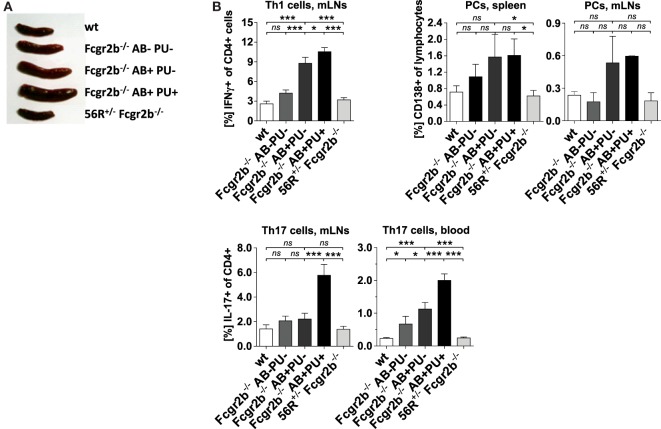
Absence of splenomegaly and no accumulation of Th1, Th17 and plasma cells (PCs) in 56R^+/−^Fcgr2b^−/−^ mice. **(A)** Spleen sizes of 5–7-month-old wt mice, Fcgr2b^−/−^ mice in the following disease states: IgG autoAb (AB)-negative and proteinuria (PU)-negative (AB^−^PU^−^), AB^+^PU^−^ and AB^+^PU^+^, and 56R^+/−^Fcgr2b^−/−^ mice. The presented organ sizes are representative of a minimum of five mice per group. **(B)** The frequency of mesenteric lymph node (mLN) CD4^+^ IFNγ^+^ Th1 cells, splenic and mLN CD138^+^PCs and mLN and blood CD4^+^ IL-17^+^ Th17 cells of 5–7-month-old wt mice (Th1 cells, mLNs, *n* = 8/PCs, spleen, *n* = 3/PCs, mLNs, *n* = 3/Th17 cells, mLNs, *n* = 8/Th17 cells, blood, *n* = 15), Fcgr2b^−/−^ mice [AB^−^PU^−^ (*n* = 12/3/3/12/10), AB^+^PU^−^ (*n* = 12/3/3/21/17) and AB^+^PU^+^ (*n* = 11/3/3/13/7)] and 56R^+/−^Fcgr2b^−/−^ mice (*n* = 12/12/3/12/26), as measured by flow cytometry. The bars indicate the mean values.

In Fcgr2b^−/−^ mice, the development of IgG2a and IgG2b autoAbs was associated with splenomegaly, increased frequencies of Th1 and PCs and IC accumulation in the kidneys (Figures 1A and 2A,B). The subsequent development of lupus nephritis (manifested by proteinuria) was additionally associated with further enhanced splenomegaly, increased frequencies of Th17 cells and infiltration of macrophages into the kidneys (Figures 1A,B and 2A,B). These findings confirm recent studies showing that the IL-17 signaling pathway is important for the development of disease in lupus-prone mice (50).

In contrast, 56R^+/−^Fcgr2b^−/−^ mice showed no signs of autoimmune inflammation (Figures 2A,B). Together these findings suggest that 56R-derived IgG autoAbs may be able to actively protect lupus-prone FcγRIIB-deficient mice from developing autoimmune inflammation.

### 56R-Derived IgG2a and IgG2b autoAbs Develop T Cell Independently in 56R^+/−^Fcgr2b^−/−^ Mice

**Figure 3:**
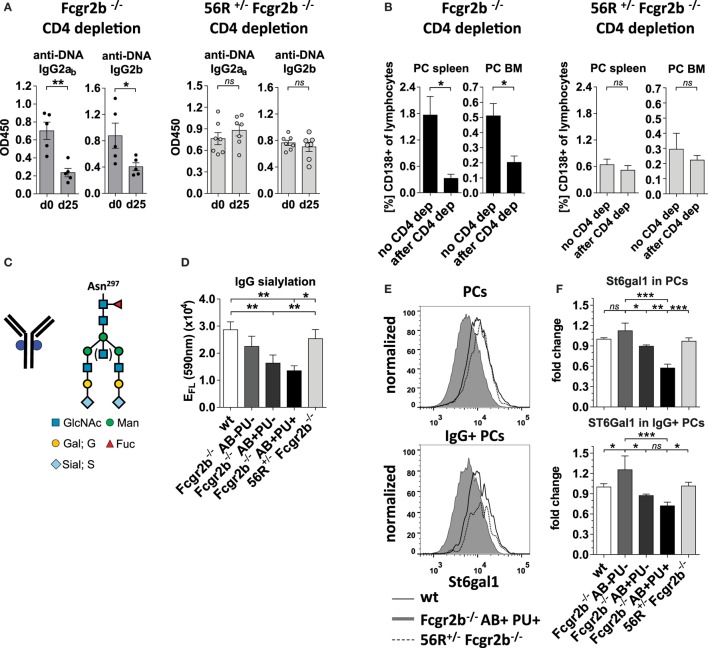
Self- and polyreactive IgG2a and IgG2b autoantibodies (autoAbs) in 56R^+/−^Fcgr2b^−/−^ mice develop T cell independently and are sialylated. **(A,B)** AutoAb-positive 5-month-old Fcgr2b^−/−^ mice or 2.5-month-old 56R^+/−^Fcgr2b^−/−^ mice received i.p. injections of anti-mouse CD4 (GK1.5) every 4 days for 25 days to deplete CD4^+^ T cells (Figure S2 in Supplementary Material). **(A)** Serum anti-DNA IgG2a_b_, IgG2b and IgG2a_a_ levels before and after CD4 depletion. **(B)** The frequencies of CD138^+^ PCs in the spleen and bone marrow (BM) of untreated (*n* = 6) versus CD4-depleted (*n* = 4) Fcgr2b^−/−^ mice, and untreated (spleen, *n* = 13; BM, *n* = 3) versus CD4 depleted (*n* = 4) 56R^+/−^Fcgr2b^−/−^ mice were analyzed *via* FACS. One representative experiment is shown. **(C)** The biantennary core of the glycan structure linked to Asn 297 in the Fc region of IgG Abs consists of four N-acetylglucosamines (GlcNAc; blue) and three mannoses (Man), which can be further modified with fucose, bisecting GlcNAc and terminal galactose (G) and sialic acid (S) residues. **(D)** The sialic acid content in serum IgG Abs of 5–6-month-old autoAb (AB)-negative and proteinuria (PU)-negative [AB^−^PU^−^ (*n* = 8), AB^+^PU^−^ (*n* = 6) and AB^+^PU^+^ (*n* = 4)] Fcgr2b^−/−^ mice and 56R^+/−^Fcgr2b^−/−^ mice (*n* = 11) compared to wt mice (*n* = 10) were analyzed with the GlykoScreen™ Sialic Acid Quantification Kit (Prozyme) (Efl: fluorescence emission at 590 nm). **(E,F)** St6gal1 protein expression in splenic total and IgG^+^ PCs of 6–7-month-old AB^−^PU^−^ (*n* = 4), AB^+^PU^−^ (*n* = 5) and AB^+^PU^+^ (*n* = 9) Fcgr2b^−/−^ mice and 56R^+/−^Fcgr2b^−/−^ mice (*n* = 6), compared to wt mice (*n* = 5). **(E)** Representative intracellular staining of St6gal1 protein expression levels in splenic PCs of wt, AB^+^PU^+^Fcgr2b^−/−^ and 56R^+/−^Fcgr2b^−/−^ mice measured by flow cytometry. **(F)** Relative median fluorescence levels of St6gal1 protein expression. Median St6gal1 protein expression levels in total or IgG^+^ PCs of three independent experiments were measured by flow cytometry and normalized (fold change) to the expression in wt controls (=1) in the respective experiments. The normalized data of three independent experiments were summarized (mean) in the graphs.

Next, we analyzed whether IgG2a and IgG2b autoAbs in 56R^+/−^Fcgr2b^−/−^ mice developed independently of T cell help as described for 56R^+/−^ mice on the C57BL/6 wt background (Figures 3A,B; Figure S2 in Supplementary Material) (47). While in Fcgr2b^−/−^ mice, depletion of CD4 cells reduced the IgG2a and IgG2b autoAb titers and enhanced frequencies of splenic and BM PCs, which is in line with reported observations (50, 60), depletion of CD4 cells in 56R^+/−^Fcgr2b^−/−^ mice with the same anti-CD4 doses failed to reduce IgG2a and IgG2b autoAb titers (Figures 3A,B; Figure S2 in Supplementary Material).

Although, it has been mentioned that autoAbs develop more or less independently of Toll-like receptor (TLR) 9 in 56R^+/−^ mice (47), it has been shown that the accumulation of class switched IgG2a and IgG2b autoAbs in 56R^+/−^Fcgr2b^−/−^ mice depends at least partially on TLR9 and particularly on MyD88 signaling (46). Hence, B cell activation and IgG2a and IgG2b class switching might take place T cell independently *via* 56R BCR and TLR/MyD88 signaling in 56R^+/−^Fcgr2b^−/−^ mice (65).

### Lupus-Resistant 56R^+/−^Fcgr2b^−/−^ Mice Generate Sialylated IgG Abs

Next we studied whether the serum IgG glycosylation differs between lupus-prone Fcgr2b^−/−^ mice and 56R^+/−^Fcgr2b^−/−^ mice. Autoimmune-prone MRL-Fas(lpr) mice and SLE patients show increased levels of pro-inflammatory agalactosylated (G0) IgGs in serum, compared to healthy controls (8, 12, 15). In line with that, serum IgG Abs from lupus-prone Fcgr2b^−/−^ mice were less sialylated, compared to wild-type controls (Figures 3C,D; Figure S1 in Supplementary Material). Reduced IgG sialylation was especially evident in Fcgr2b^−/−^ mice that developed signs of lupus nephritis (proteinuria and kidney inflammation) (Figure 3D). In contrast, serum IgG sialylation in 56R^+/−^Fcgr2b^−/−^ mice was comparable to that of healthy wild-type animals (Figure 3D).

Notably, the protein expression level of the alpha2,6-sialyltransferase 1 (St6gal1), which is responsible for terminal sialylation of IgG Fc glycan (24, 29–31), was reduced in total and IgG-switched PCs (IgG^+^PC) of Fcgr2b^−/−^ mice with nephritis, but not in PCs of 56R^+/−^Fcgr2b^−/−^ mice (Figures 3E,F). These data are consistent with our recent findings that T cell independent B cell activation leads to the development of PCs expressing high levels of St6gal1 and producing sialylated IgG Abs (30).

### Transfer of ICs Containing Sialylated IgG Abs Inhibit Nephritis in Fcgr2b^−/−^ Mice

**Figure 4:**
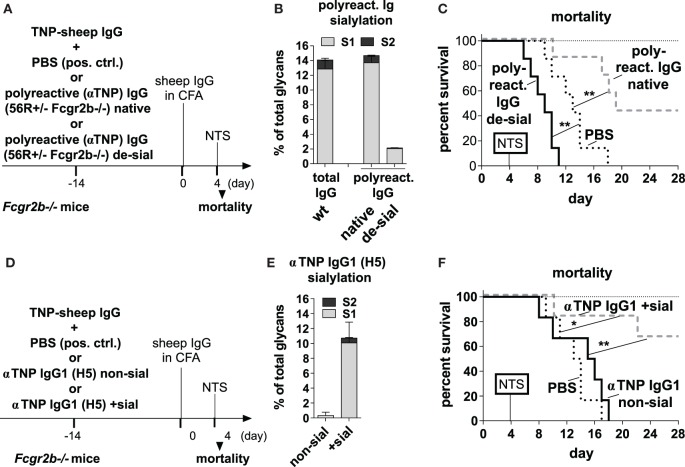
Transfer of sialylated polyreactive IgG antibodies (Abs) derived from 56R^+/−^Fcgr2b^−/−^ mice or administration of sialylated antigen-specific monoclonal IgG Abs reduces nephritis-induced mortality in Fcgr2b^−/−^ mice. **(A–C)** Transfer of sialylated polyreactive IgG Abs from 56R^+/−^Fcgr2b^−/−^ mice reduces nephritis-induced mortality. **(A)** Graphical representation of the experimental strategy. Fcgr2b^−/−^ mice received ICs containing 100 µg of TNP-sheep IgG and 200 µg of either sialylated (native) or sialidase-treated de-sialylated (de-sial) polyreactive IgG Abs derived from 2–3-month-old 56R^+/−^Fcgr2b^−/−^ mice. The positive control (pos. ctrl.) group was treated with PBS and TNP-sheep IgG only. After 14 days, nephritis was induced by injection of sheep IgG in CFA and subsequent intravenous injection of sheep anti-glomerular basement membrane nephrotoxic serum (NTS) 4 days later. **(B)** Fc sialylation of purified total serum IgG Abs from wt mice and purified polyreactive IgG Abs from 56R^+/−^Fcgr2b^−/−^ mice before and after sialidase treatment was determined through EndoS-treatment and MALDI-TOF mass spectrometry (MS) (percentage of glycans with one or two sialic acid residues: S1 and S2; Figure S1 in Supplementary Material). **(C)** Kaplan-Meier survival analysis of the indicated groups. **(D–F)** Application of sialylated antigen-specific monoclonal IgG Abs reduces nephritis-induced mortality. **(D)** Graphical representation of the experimental strategy as described in **(A)**. Fcgr2b^−/−^ mice were treated with ICs containing 100 µg of TNP-sheep IgG and 100 µg of either native non-sialylated (αTNP murine IgG1 non-sial) or *in vitro* sialylated (αTNP IgG1 +sial) monoclonal anti-TNP murine IgG1 (clone H5) Abs. After 14 days, nephritis was induced as described above. **(E)** Fc sialylation of the monoclonal anti-TNP murine IgG 1 Abs (clone H5) before and after *in vitro* sialylation was determined through EndoS-treatment and MALDI-TOF MS (the percentage of one or two sialic acid residues coupled to the glycan: S1, S2; Figure S1 in Supplementary Material). **(F)** Kaplan–Meier survival analysis for the indicated groups. One representative experiment out of two independent experiments is shown.

To address such a possible ameliorating effect, we next tested whether ICs containing sialylated IgG autoAbs from 56R^+/−^Fcgr2b^−/−^ mice can directly attenuate the onset of nephritis in an induced nephritis model (53). We purified polyreactive TNP-binding (Figure S2 in Supplementary Material) IgG Abs from sera of 56R^+/−^Fcgr2b^−/−^ mice and generated ICs with TNP-coupled sheep (TNP-sheep) IgG Abs using either the purified native (sialylated) or *in vitro* sialidase-treated (de-sialylated) polyreactive IgG Abs (Figures 4A,B). The ICs were then transferred to Fcgr2b^−/−^ mice and, 2 weeks later, the nephritis was induced by injecting TNP-sheep IgG Abs in CFA followed by i.v. injection of sheep anti-GBM NTS 4 days later (Figure 4) (56).

The ICs containing de-sialylated polyreactive IgG Abs increased nephritis-induced mortality when compared to a positive control (Figure 4C). In contrast, the ICs containing native sialylated polyreactive IgG Abs attenuated nephritis-induced mortality in Fcgr2b-/- mice (Figure 4C). Similarly, ICs containing *in vitro* sialylated anti-TNP monoclonal murine IgG1 (clone H5) Abs, but neither native non-sialylated anti-TNP monoclonal nor sialylated antigen-unspecific monoclonal murine IgG1 Abs, reduced mortality in this nephritis model (Figures 4D–F; Figure S3 in Supplementary Material). In summary, these results showed that only antigen-specific sialylated IgG Abs were able to attenuate disease development.

### Sialylated Collagen-Specific IgG autoAbs Attenuate Autoimmune Inflammation in the CIA Model Independent of FcγRIIB

In order to see whether these sialylation dependent attenuating effects are detectable in a broader spectrum of autoimmune disease models, we further analyzed whether sialylated IgG autoAbs are able to attenuate autoimmune pathology and inflammation also in the collagen type II-induced arthritis (CIA) model. In contrast to earlier studies (24) we chose FcγRIIB-deficient mice for our experiments (66), because the observed inhibitory effect of sialylated IgG autoAbs in the former experiments was FcγRIIB-independent.

**Figure 5:**
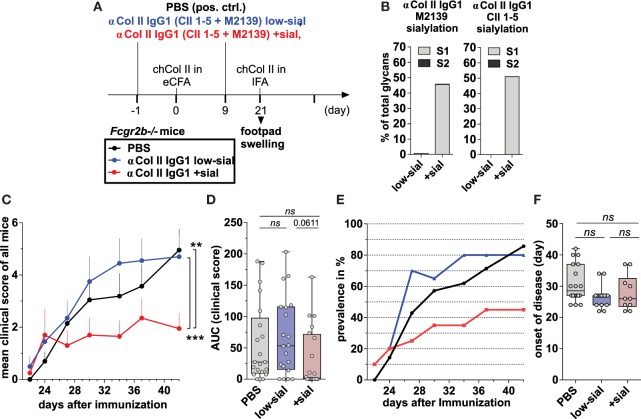
Sialylated collagen type II (Col II)-reactive monoclonal IgG antibodies (Abs) suppress collagen-induced arthritis (CIA). **(A)** Graphical representation of the experimental strategy. CIA was induced in Fcgr2b^−/−^ mice by subcutaneous injection of Col II in enriched CFA (eCFA) and subsequent challenge with Col II in incomplete Freund’s adjuvant (IFA) on day 21. One day before and 9 days after the first immunization, the mice received 100 µg of either low-sialylated (low-sial) or *in vitro* galactosylated plus sialylated (+sial) anti-Col II murine IgG1 Abs (clones M2139 and CII 1–5; 50 µg each) The positive control group received PBS instead of Abs. **(B)** Fc sialylation of native (low-sial) and *in vitro* galactosylated plus sialylated (+ sial) anti-Col II M2139 and CII 1–5 murine IgG1 Abs measured by EndoS-treatment and MALDI-TOF mass spectrometry (MS) (percentage of glycans with one or two sialic acid residues: S1 and S2; Figure S1 in Supplementary Material). **(C–F)** Combined clinical data of two independent CIA experiments (PBS: *n* = 21; low-sial: 21; +sial; *n* = 20). Foot swelling was scored on the indicated days from 0 to 3 per foot resulting in a maximal clinical score of 12 per mouse. The **(C)** mean clinical score of all mice and the **(E)** prevalence (percentage of affected animals with a score > 0) are shown for all groups on the indicated days. **(D)** The area under the curve (AUC) of the clinical score over the time was calculated for each mouse. AUC = 0 indicates that the animal never developed foot swelling (mice with score 0: PBS: *n* = 3; low-sial: *n* = 4; +sial: *n* = 10). **(F)** The day of disease onset shown only of the mice that developed foot swelling (score > 0) during the experiment. Differences in disease evolution (C) were analyzed using two-way ANOVA.

We produced two monoclonal murine Col II-reactive murine IgG1 Abs (clones M2139 and CII 1–5; **57, 58**) in a native, very low-sialylated form and then generated sialylated forms of these Abs by *in vitro* galactosylation and sialylation (Figures 5A,B; Figures S1 and S5 in Supplementary Material), which do not affect antigen reactivity (Figure S5 in Supplementary Material). CIA was induced *via* s.c. injection of chicken Col II in enriched CFA and challenged with Col II in IFA 3 weeks later (Figure 5A).

100 µg of the Col II-reactive IgG1 Abs (either the low-sialylated or the sialylated form) were administered twice –1 day before and 9 days after the first immunization (Figure 5A). Foot swelling (clinical scores of 0–3 per foot with a maximum clinical score of 12 per mouse) was used as the marker to assess the CIA reaction.

The sialylated, but not the low-sialylated, Col II-reactive IgG Abs significantly reduced the mean clinical score of foot swelling, as compared to a PBS-treated control group (Figures 5C,D). In detail, only about 50% of the mice treated with the sialylated Col II-specific IgG1 Abs started to develop foot swelling (clinical score > 0 per mouse), whereas more than 80% of the mice treated with low-sialylated Col II-specific IgG Abs or with PBS developed foot swelling (Figures 5D,E). No significant differences in the timing of the disease onset were observed between the groups (Figure 5F). Together, these data are consistent with recent findings that sialylated Col II-specific IgG1 Abs can attenuate CIA in DBA/1 mice (24). Their studies further showed that the suppressive effect of sialylated IgG autoAbs was autoantigen-specific; an antigen-unspecific sialylated IgG1 Ab failed to attenuate CIA in their model (24). Here, we further show, that the attenuation of CIA with sialylated IgG autoAbs is independent of FcγRIIB.

Furthermore, the effect of 100 µg of the different sialylated Col II-specific IgG1 Abs was compared to the effect of high (50 mg; approximately 2 g/kg) and low (100 µg; approximately 4 mg/kg) doses of IVIG on the induction of CIA and particular inflammatory T and B cell responses in the CIA model (Figure S5 in Supplementary Material). We found that high doses of IVIG attenuated the mean clinical score and the prevalence of CIA such as low amounts (1/500 compared to high dose IVIG) of sialylated IgG autoAbs (Figure S5 in Supplementary Material), whereas administration of equal amounts of non-specific (sialylated) IgG (low doses of IVIG) was insufficient to alleviate autoimmune inflammation. Random analysis of ankle sections by histology H&E and anti-CD3 staining showed no differences between mice from different groups with identical foot scores (Figure S5 in Supplementary Material and data not shown).

**Figure 6:**
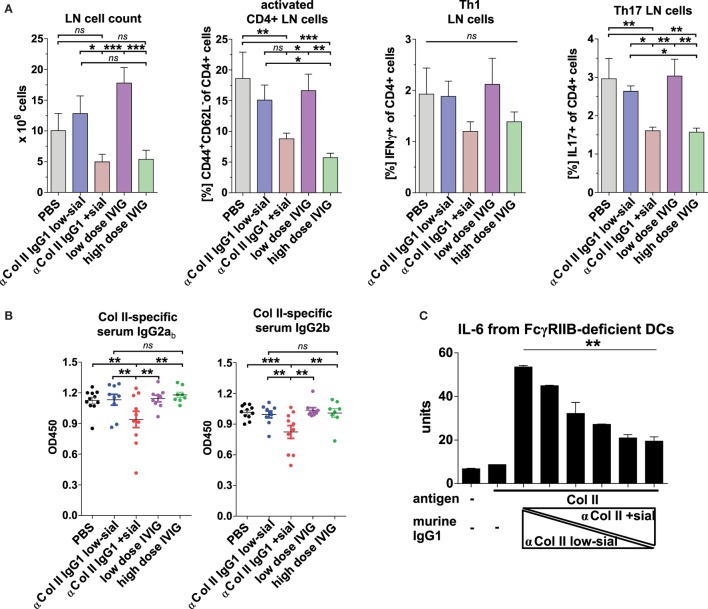
Sialylated collagen type II (Col II)-reactive monoclonal IgG antibodies (Abs) reduce the accumulation of proinflammatory Th17 cells and IgG autoAbs. Collagen-induced arthritis (CIA) was induced in Fcgr2b^−/−^ mice as described in Figure 5 and Figure S5C in Supplementary Material. One day before and 9 days after the first immunization, the mice received 100 µg of either low-sialylated (low-sial; *n* = 10) or *in vitro* galactosylated plus sialylated (+sial; *n* = 9) anti-Col II murine IgG1 Abs (clones M2139 and CII 1–5; 50 µg each) or high dose (50 mg; *n* = 10) or low dose (100 µg; *n* = 10) of intravenous immunoglobulin (IVIG) (Figure S5B in Supplementary Material). The positive control group received PBS instead of Abs (*n* = 10). **(A)** Pooled popliteal and brachial lymph nodes (LN) of each mouse from the indicated groups were analyzed on day 47 to determine total cell counts and the frequencies of activated CD4^+^CD44^+^CD62L^−^ T cells, CD4^+^IFNγ^+^ Th1 cells and CD4^+^IL-17^+^ Th17 cells. **(B)** Col II-reactive IgG2a_b_ and IgG2b serum Ab levels measured on day 42 by enzyme-linked immunofluorescence assay (ELISA). One representative experiment out of two independent experiments is shown. **(C)** Sialylation of Col II-specific IgG1 Abs inhibits IL-6 secretion by dendritic cells (DCs). Bone marrow-derived DCs from Fcgr2b^−/−^ mice were cultured in the presence of ICs containing 1 µg of Col II and 4 µg of different ratios of low-sialylated (< 1% sialylation; Figure 5B) and *in vitro* galactosylated and sialylated (46% sialylation; Figure 5B) Col II-specific IgG1 Abs (clone M2139; % of sialylation from left to right: <1, 3, 6, 12, 23, 46%). The IL-6 concentration in the supernatant was analyzed after 36 h *via* ELISA. The presented data are representative of four independent experiments.

Also, high, but not low, doses of IVIG and sialylated, but not low-sialylated, Col II-reactive IgG autoAbs reduced by trend the accumulation of Th1 cells and significantly the accumulation of inflammatory Th17 cells, which are known for their important role also in the pathogenesis of CIA (Figure 6A) (67–71). Interestingly, unlike sialylated Col II-specific IgG1 Abs, the high doses of IVIG failed to inhibit the generation of Col II-specific IgG2 autoAbs (Figure 6B) suggesting a different or additional mechanism of low doses of sialylated Col II-specific IgG Abs as compared to high doses of IVIG.

We could not detect a significant influence of sialylated or low-sialylated anti-Col II IgG Abs on the frequency of total Foxp3^+^ regulatory CD4 T (Treg) cells in the CIA model (data not shown). However, we observed an increase in antigen-specific Foxp3^+^Treg frequencies and a tendency toward a reduction of antigen-specific CD4 T cell proliferation with sialylated IgG Abs as compared to low-sialylated IgG Abs in a transfer model with OVA-specific (OT-II) CD4 T cells in C57BL/6 wt and Fcgr2b^−/−^ mice (Figure S6 in Supplementary Material).

Also, the induction of OVA-specific Foxp3^+^Tregs by sialylated monoclonal TNP-specific IgG Abs seemed to be antigen-specific, as mice immunized with OVA (in contrast to TNP-OVA) failed to elicit a comparable increase in Foxp3^+^Treg frequencies (Figures S6D,E in Supplementary Material).

These data further suggested an effect of sialylated antigen-specific IgG Abs on the adaptive immune response through an FcγRIIB-independent mechanism.

Matured dendritic cells (DCs) that produce inflammatory cytokines are a prerequisite for induction of inflammatory T and B cell responses and there is evidence that ICs containing sialylated IgG Abs can inhibit DC activation (29). We assessed how IgG sialylation modulates the capacity of ICs to suppress IL-6 production, a cytokine critical for Th17 generation (72, 73) and CIA development (70, 71, 74, 75), by BM-derived FcγRIIB-deficient DCs *in vitro* (Figure 6C). IL-6 secretion induced by treating DCs with ICs containing asialylated Col II-reactive IgG1 autoAbs was dramatically reduced by adding sialylated IgG Abs (Figure 6C).

In summary, the data suggest that ICs containing antigen and sialylated antigen-specific IgG Abs can influence DC activation and thereby regulate antigen-specific T and finally B cell responses.

## Discussion

Fc glycosylation of IgG molecules regulates their effector functions and thereby may critically contribute to development of autoimmune pathology. On the one hand, agalactosylated IgG autoAbs are associated with severity of autoimmune disorders, such as RA and SLE (3–25), and able to induce disease symptoms in mouse models of RA (9, 24). On the other hand, the presence of autoAbs in sera of many healthy humans (76–79) implies that additional factors, including Fc glycosylation, seem crucial for rendering autoAbs pathogenic. For instance, RA patients develop IgG autoAbs long before clinical symptoms (21, 80), and the glycosylation patterns of these early autoAbs are less inflammatory compared to IgG Abs detected in patients with the manifested disease (21, 25).

Another unexplored possibility is that anti-inflammatory sialylated autoAbs not only lack pathogenic activity, but may be responsible for inducing immune tolerance to autoantigens in healthy animals and humans. Indeed, we have recently demonstrated that T-cell-independent antigens promote generation of immunosuppressive sialylated IgG molecules (30), whereas T-cell-dependent immunizations can induce antigen-specific IgG Abs with pro- or anti-inflammatory glycosylation patterns depending on the co-stimuli (29, 30). Our present study further suggests that sialylated IgG2a and IgG2b autoAbs produced by 56R BCR-expressing B cells in a T-cell-independent manner are able to attenuate development of nephritis in lupus-prone FcγRIIB-deficient mice. These findings therefore would extend the existing knowledge that B cells regulate immune responses and inhibit autoimmune pathology *via* secretion of immunosuppressive cytokines, such as IL-10 and IL-35 (81).

Antigen specificity of sialylated IgG Abs may play a critical role in exerting their anti-inflammatory effect. Indeed, the transfer of ICs containing sialylated polyreactive IgG autoAbs from 56R^+/−^Fcgr2b^−/−^ mice or sialylated antigen-specific monoclonal IgG Abs attenuated the development of the induced nephritis (Figure 4), whereas antigen-unspecific sialylated monoclonal IgG Abs failed to reach the inhibitory potential of antigen-specific sialylated IgG Abs.

In the arthritis model, sialylated Col II-specific IgG Abs reduced arthritis symptoms (Figure 5), whereas administration of equal amounts of non-specific (sialylated) IgG (low dose of IVIG) was insufficient to alleviate autoimmune inflammation. These data are well consistent with recent reports showing that sialylated Col II-specific IgG1 Abs can inhibit CIA in DBA/1 mice (24). Importantly, Ohmi et al. demonstrated that the inhibitory effect of sialylated IgG autoAbs in the CIA model is autoantigen-specific, since non-specific sialylated IgG1 failed to suppress CIA (24). We cannot exclude, however, that the suppressive effects of sialylated IgG autoAbs observed here are only partially mediated in an antigen specific manner.

Mouse studies that used IVIG and sialylated Fc fragments suggest that antigen specificity is not essential for the anti-inflammatory action of the sialylated subfraction of IVIG (35). By comparing the effects of high doses of (sialylated) IVIG and sialylated collagen-specific IgG Abs, our data suggest that antigen specificity might significantly enhance the capacity of sialylated Abs to inhibit immune reactions. In accordance, we found that small amounts of collagen-specific sialylated IgG1 Abs, but not high doses of IVIG, were able to inhibit the development of IgG2 autoAbs in the CIA model. Since antigen specificity is necessary for IC formation, antigen-specific sialylated IgG Abs might inhibit IgG autoAb production *via* an alternative pathway that potentially requires IC formation.

Extensive evidence suggest that generation of Th17 cells plays a crucial role in pathogenesis of many autoimmune disorders and mouse models of SLE and CIA are dependent on IL-17 (50, 67, 70, 71). Moreover, IL-17 is necessary for development of pathogenic G0 IgG Abs (30). IL-6 skews T cell differentiation toward IL-17A-producing Th17 cells, suppressing the generation of Foxp3^+^Treg cells (72, 73). In line, we showed that sialylation of IgG autoAbs reduces IL-6 production by DCs *in vitro* and Th17 cell accumulation in autoimmune models. Moreover, we observed that only the formation of sialylated IgG ICs increases the frequencies of antigen-specific Foxp3^+^Treg cells in the OT-II^+^T cell transfer model.

In summary, we suppose that ICs containing autoantigen-specific sialylated IgG Abs influence inflammatory DC activation and IL-6 production in a FcγRIIB-independent manner and thereby downregulate Th17 generation, formation of pathogenic G0 autoAbs and, hence, alleviate clinical signs of autoimmune pathology.

## Ethics Statement

All of the mice were bred and maintained at the German Rheumatism Research Center in Berlin or the University of Lübeck, Germany and all experiments were conducted with the approval of and in accordance with regulatory guidelines and ethical standards set by both institutions and the Ministry of Berlin or Schleswig-Holstein, Germany.

## Author Contributions

YCB, JR and MMMM conducted key experiments. SE and FKML performed the chicken collagen induced arthritis mouse experiments. ADS, DB, AKL, AW, G-ML, JP, JH, MS, CH, VH, CTS, CMO and AL performed some of the *in vivo* and *in vitro* experiments. YCB, DB and VB performed IgG glycan analysis. ME coordinated and supervised the experiments and wrote the manuscript.

## Conflict of Interest Statement

The authors declare that the research was conducted in the absence of any commercial or financial relationships that could be construed as a potential conflict of interest.

## Supplementary Material

Figure S1 EndoS-released glycan structures. **(A)** The biantennary core of the glycan structure linked to Asn 297 in the Fc region of IgG antibodies (Abs) consists of four N-acetylglucosamines (GlcNAc; blue) and three mannoses (Man), which can be further modified with fucose, bisecting GlcNAc and terminal galactose (G) and sialic acid [S; human (purple) – N-acetylneuraminic acid (Neu5Ac) or murine (light-blue) – N-glycolylneuraminic acid (Neu5Gc)] residues. MALDI-TOF mass spectrometry (MS) of N-glycans linked to Asn 297 at IgG Fc fragments was performed through EndoS-treatment; the endoglycosidase S (EndoS) cleavage site is indicated with an arrow. **(B)** Possible human and murine Fc glycan structures released from Asn 297 using EndoS. The patterns include glycan structures with human (blue) sialic acid (Neu5Ac) residues or murine (red) sialic acid residues (Neu5Gc). The numbers represent the molecular mass (*m*/*z*) of the possible Fc glycan structures (permethylated) released upon EndoS treatment.

Figure S2 Early development of self- and polyreactive IgG2a and IgG2b autoAbs in 56R^+/−^Fcgr2b^−/−^ mice is T cell independent. These data are part of the analyses described in Figures 1–3. **(A)** Anti-nucleosome (nuc) and **(B)** anti-TNP (polyreactive) IgG2a_b_, IgG2b or IgG2a_a_ serum subclass antibodies (Abs) of 5–7-month-old wt and Fcgr2b^−/−^ mice and 2- or 6-month-old 56R^+/−^Fcgr2b^−/−^ mice were determined *via* enzyme-linked immunofluorescence assay (ELISA). Each data point represents an individual animal. Bars indicate mean values. **(C,D)** AutoAb-positive 5-month-old Fcgr2b^−/−^ mice or 2.5-month-old 56R^+/−^Fcgr2b^−/−^ mice were treated every 4 days (d) with i.p. injections of anti-mouse CD4 (GK1.5) to deplete CD4^+^ T cells for 25 days. **(C)** The efficacy of CD4^+^TCRbeta^+^ cell depletion *via* anti-CD4 Ab treatment was monitored through FACS analysis of peripheral blood samples from C57BL/6 wt mice on days 2 and 5 after a single injection of 250 µg of anti-CD4. **(D)** Serum anti-nucleosome (nuc) IgG2a_b_, IgG2b and IgG2a_a_ autoAb levels before and after CD4 depletion were determined *via* ELISA. Data from the same representative experiment as described in Figures 3A,B are shown. **(E)** Representative intracellular staining of St6gal1 protein expression levels in splenic plasma cells (PCs) of a wt and AB^+^PU^+^Fcgr2b^−/−^ mouse and an isotype control staining of the same AB^+^PU^+^Fcgr2b^−/−^ mouse analyzed by flow cytometry.

Figure S3 Sialylated antigen-specific IgG antibodies (Abs) protect significant better from mortality in the nephrotoxic nephritis model than sialylated IgG Abs with an irrelevant specificity. **(A)** The data in **(A)** are also part of the experiment described in Figures 4D–F. TNP-specific enzyme-linked immunofluorescence assay (ELISA); the Fc glycosylation pattern of anti-TNP murine IgG1 Abs (clone H5) has no influence on TNP recognition. **(B)** Graphical representation of the experimental strategy. C57BL/6 wt mice received 100 µg of TNP-sheep IgG and 100 µg of either sialylated antigen-specific anti-TNP murine IgG1 (clone: H5; H5 + sial; *n* = 6) or sialylated antigen-unspecific anti-Thy1.1 murine IgG1 (clone: OX-7; OX-7 + sial; *n* = 4) Abs. After 14 days, nephritis was induced by injection of sheep IgG in complete Freund’s adjuvant (CFA) and subsequent intravenous treatment with sheep anti-glomerular basement membrane nephrotoxic serum (NTS) 4 days later. **(C)** Fc sialylation of the monoclonal anti-TNP murine IgG 1 (clone H5) and anti-Thy1.1 murine IgG1 (clone OX-7) Abs was determined through EndoS-treatment and MALDI-TOF mass spectrometry (MS) (percentage of glycans with one or two sialic acid residues: S1 and S2). **(D)** Kaplan–Meier survival analysis of the indicated groups. One representative experiment is shown.

Figure S4 Sequences and cloning of the CII-reactive monoclonal murine IgG1 antibodies used in the experiments shown in Figures 5 and 6 and Figure S5 in Supplementary Material. The start and stop codons are colored in red. The restriction sites used for cloning are highlighted in yellow. The first box (blue) represents the leader sequence. The second box (yellow) shows the variable VDJ or VJ sequence whereas the third box (gray) is the constant IgG1-Fc or kappa chain sequence, respectively. **(A) Col II-reactive (clone M2139) murine IgG1 heavy chain** (accession number: MH208236). The 43 bold and underlined bases behind the leader sequence were missing in the original sequence description (NCBI Z72462) (57) and completed here by the J558.2.88 (NCBI BN000872) sequence because of its highest homology to the original sequence observed after NCBI, IgBlast alignment. The C57BL/6 IgG1 heavy chain constant region starts two bases in front of the *SalI* restriction site. Because of the introduction of the *SalI* restriction site, the constant IgG1 heavy chain sequence starts with the amino acids (A)STT… instead of (A)KTT…, which had no functional influence (60). **(B) Col II-reactive (clone M2139) murine kappa chain** (accession number: MH208237). The first 27 bold and underlined bases behind the leader sequence were missing in the original sequence description (NCBI Z72463) (57) and were completed here by the 21-1 (NCBI X16955) sequence because of its highest homology to the original sequence observed after NCBI, IgBlast alignment. The IGKJ2 sequence in the original sequence description was incomplete and completed here with bold and underlined letters representing the IGKJ2 sequence (accession number V00777) identified on the ImMunoGeneTics (IMGT) Marie-Paule homepage (http://www.imgt.org). The C57BL/6 constant light chain region starts with the *BsiWI* restriction site. Because of the introduction of the *BsiWI* restriction site, the constant kappa light chain sequence starts with the amino acids (R)TDA… instead of (R)ADA…, which had no functional influence (60). **(C) Col II-reactive (clone CII 1-5) murine IgG1 heavy chain** (accession number: MH208238). The IGHJ2 sequence in the original sequence description (NCBI MMU69538) (58) was incomplete and completed here with bold and underlined letters representing the IGHJ2 sequence (accession number V00770) identified on the ImMunoGeneTics (IMGT) Marie-Paule homepage (http://www.imgt.org). The C57BL/6 IgG1 heavy chain constant region starts two bases in front of the *SalI* restriction site. Because of the introduction of the *SalI* restriction site, the constant IgG heavy chain sequence starts with the amino acids (A)STT… instead of (A)KTT…, which had no functional influence (60). **(D) Col II-reactive (clone CII 1-5) murine kappa chain** (accession number: MH208239). The four bold and underlined bases behind the leader sequence were corrected from the original sequence description (NCBI MMU69539) (58) because of its obvious mismatching from the highly homologous 12-44 (NCBI AJ235955) sequence observed after NCBI, IgBlast alignment probably resulting by sequencing/analyzing mistakes. The exchanges were from g to c, c to a, c to g and a to g. The IGKJ1 sequence in the original sequence description was incomplete and completed here with bold and underlined letters representing the IGKJ1 sequence (accession number V00777) identified on the ImMunoGeneTics (IMGT) Marie-Paule homepage (http://www.imgt.org). The C57BL/6 constant light chain region starts with the *BsiWI* restriction site. Because of the introduction of the *BsiWI* restriction site the constant kappa light chain sequence starts with the amino acids (R)TDA… instead of (R)ADA…, which had no functional influence (60).

Figure S5 Low doses of sialylated Col II-reactive monoclonal IgG antibodies (Abs) and high doses of intravenous immunoglobulin (IVIG) attenuate collagen-induced arthritis (CIA). These data are parts of the experiments described in Figures 5 and 6. **(A)** Col II reactivities of native low-sialylated (low-sial) and *in vitro* galactosylated plus sialylated (+sial) M2139 and CII 1-5 IgG1 Abs and of an antigen-unspecific, TNP-specific murine IgG1 hybridoma Ab (clone H5; negative control) (52) were determined through ELISA. **(B)** Graphical representation of the experimental strategy used in Figure 6. CIA was induced in Fcgr2b^−/−^ mice as described in Figure 5A. One day before and 9 days after the first immunization the mice received 100 µg of either non-sialylated (low-sial; *n* = 10) or *in vitro* galactosylated plus sialylated (+sial; *n* = 9) anti-Col II murine IgG1 Abs (clones M2139 and CII 1-5; 50 µg each) or high dose (50 mg; *n* = 10) or low dose (100 µg; *n* = 10) IVIG. The positive control group received PBS instead of Abs (*n* = 10). **(C)** Foot swelling was scored on the indicated days from 0 to 3 per foot resulting in a maximal clinical score of 12 per mouse. The mean clinical score of all mice and the prevalence (percentage of affected animals with a score > 0) are shown for all groups on the indicated days. **(D)** Representative foot ankle sections of representative mice with CIA score 0 (left; healthy mouse) or score 2 (right; mouse with a swollen ankle) were stained with hematoxylin and eosin (H&E) or anti-CD3. One representative experiment out of two independent experiments is shown. Differences in disease evolution were calculated using two-way ANOVA.

Figure S6 Application of sialylated antigen-specific murine IgG1 antibodies (Abs) enhances the development of antigen-specific Foxp3^+^Tregs. **(A)** Graphical representation of the experimental strategy. On day −1, C57BL/6 wt or Fcgr2b^−/−^ mice were treated intravenous with 3 × 10^7^ labeled splenocytes from OT-II^+/−^ × Thy1.1^+/−^ mice and subsequently treated i.p. with 90 µg of low-sialylated (low-sial) or *in vitro* galactosylated plus sialylated (sial) monoclonal murine anti-TNP IgG1 Abs (clone H5) or no Ab. On day 0, mice were immunized i.p. with 30 µg of TNP(5)-OVA or OVA (antigen-unspecific control) in enriched CFA (eCFA) and on day 4, mice were sacrificed for flow cytometry analysis of mesenteric lymph node (mLN) cells and splenocytes. **(B)** Fc sialylation of low-sialylated or *in vitro* galactosylated plus sialylated anti-TNP IgG1 Abs (H5) was determined through EndoS-treatment and MALDI-TOF mass spectrometry (MS) (percentage of glycans with one or two sialic acid residues: S1, S2; Figure S1 in Supplementary Material). **(C)** Representative Foxp3 expression analysis of gated, proliferating (as a marker for antigen-specific activation) OT-II^+/−^ × Thy1.1^+/−^CD4^+^ mLN cells of all three groups treated with TNP-OVA by flow cytometry on day four. **(D,E)** Foxp3^+^cell frequencies of transferred proliferating OT-II^+/−^ × Thy1.1^+/−^ mLN and spleen cells from C57BL/6 wt mice after immunization with **(D)** TNP-OVA or **(E)** OVA in eCFA as determined by flow cytometry. **(F)** Percentage of proliferating OT-II^+/−^ × Thy1.1^+/−^ of all CD4^+^ spleen cells from C57BL/6 wt mice after immunization with TNP-OVA in eCFA. One representative out of two independent experiments is shown. **(G)** Foxp3^+^ cell frequencies of transferred proliferating OT-II^+/−^ × Thy1.1^+/−^ mLN and spleen cells from Fcgr2b^−/−^ mice after immunization with TNP-OVA in eCFA. One representative out of two independent experiments is shown.

## Abbreviations

Ab, antibody; autoAb, autoantibody; BCR, B cell receptor; CFA, complete Freund’s adjuvant; CIA, collagen-induced arthritis; DC, dendritic cell; eCFA, enriched complete Freund’s adjuvant; IC, immune complex; IFA, incomplete Freund’s adjuvant; OVA, ovalbumin; PC, plasma cell; RA, rheumatoid arthritis; SLE, systemic lupus erythematosus; St6gal1, beta-galactoside alpha2,6-sialyltransferase 1; TCR, T cell receptor; TNP, 2,4,6-trinitrophenyl.
