# Supplementary material for: Sialylated Autoantigen-Reactive IgG Antibodies Attenuate Disease Development in Autoimmune Mouse Models of Lupus Nephritis and Rheumatoid Arthritis
Source: Front Immunol. 2018 Jun 6;9:1183. doi: 10.3389/fimmu.2018.01183 (PMC5997785; doi:10.3389/fimmu.2018.01183)
Supplement: Figure S1 — EndoS-released glycan structures. (A) The biantennary core of the glycan structure linked to Asn 297 in the Fc region of IgG antibodies (Abs) consists of four N-acetylglucosamines (GlcNAc; blue) and three mannoses (Man), which can be further modified with fucose, bisecting GlcNAc and terminal galactose (G) and sialic acid [S; human (purple) – N-acetylneuraminic acid (Neu5Ac) or murine (light-blue) – N-glycolylneuraminic acid (Neu5Gc)] residues. MALDI-TOF mass spectrometry (MS) of N-glycans linked to Asn 297 at IgG Fc fragments was performed through EndoS-treatment; the endoglycosidase S (EndoS) cleavage site is indicated with an arrow. (B) Possible human and murine Fc glycan structures released from Asn 297 using EndoS. The patterns include glycan structures with human (blue) sialic acid (Neu5Ac) residues or murine (red) sialic acid residues (Neu5Gc). The numbers represent the molecular mass (m/z) of the possible Fc glycan structures (permethylated) released upon EndoS treatment. [file Presentation_1.PDF]

A

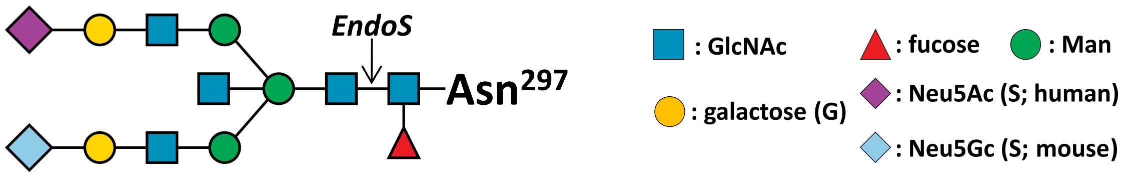


B

**MALDI-TOF: Human and mouse IgG Fc glycan structures (EndoS)**

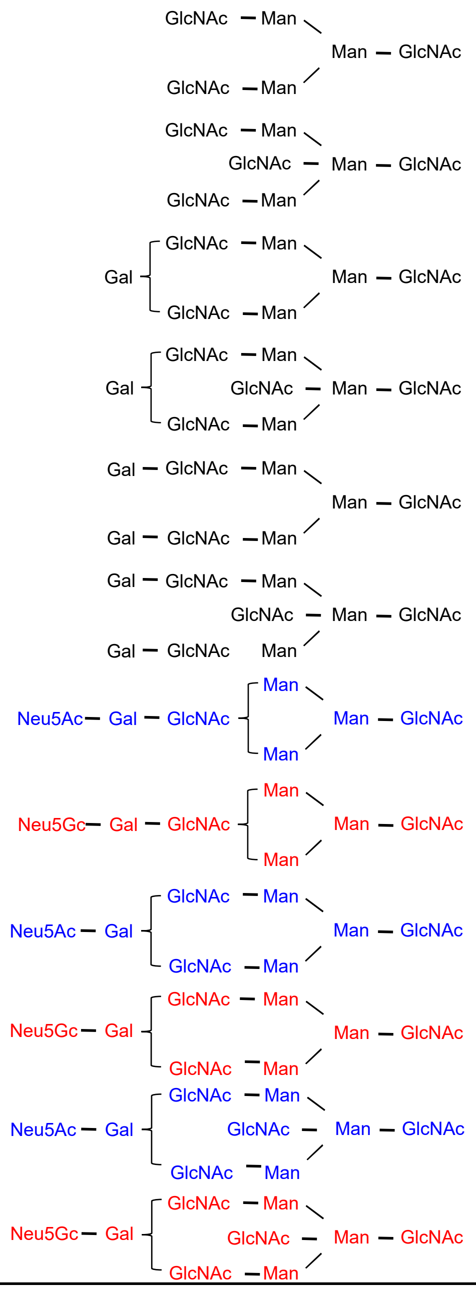


| mass (m/z) | abbreviation |
|---|---|
| 1417 | G0 |
| 1662 | G0 |
| 1621 | G1 |
| 1866 | G2 |
| 1825 | G2 |
| 2070 | G2 |
| 1737 | G1 S1 (human) |
| 1767 | G1 S1 (mouse) |
| 1982 | G1 S1 |
| 2012 | G1 S1 |
| 2227 | G1 S1 |
| 2257 | G1 S1 |

Fig. S1

MALDI-TOF: Human and mouse IgG Fc glycan structures (EndoS) mass (m/z) abbreviation

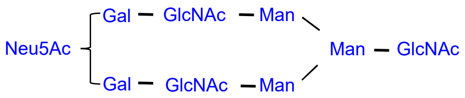


2186 G2 S1

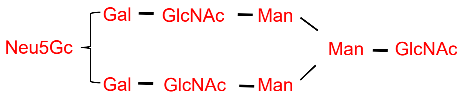


2216 G2 S1

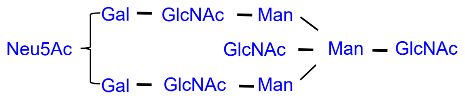


2431 G2 S1

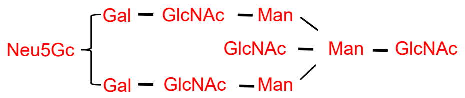


2461 G2 S1

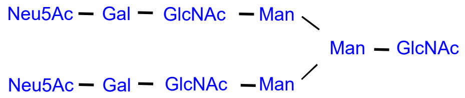


2547 G2 S2

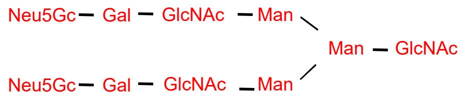


2607 G2 S2

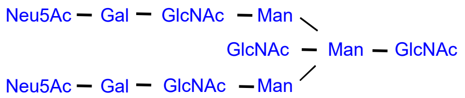


2792 G2 S2

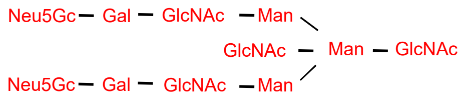


2852 G2 S2

Fig. S1

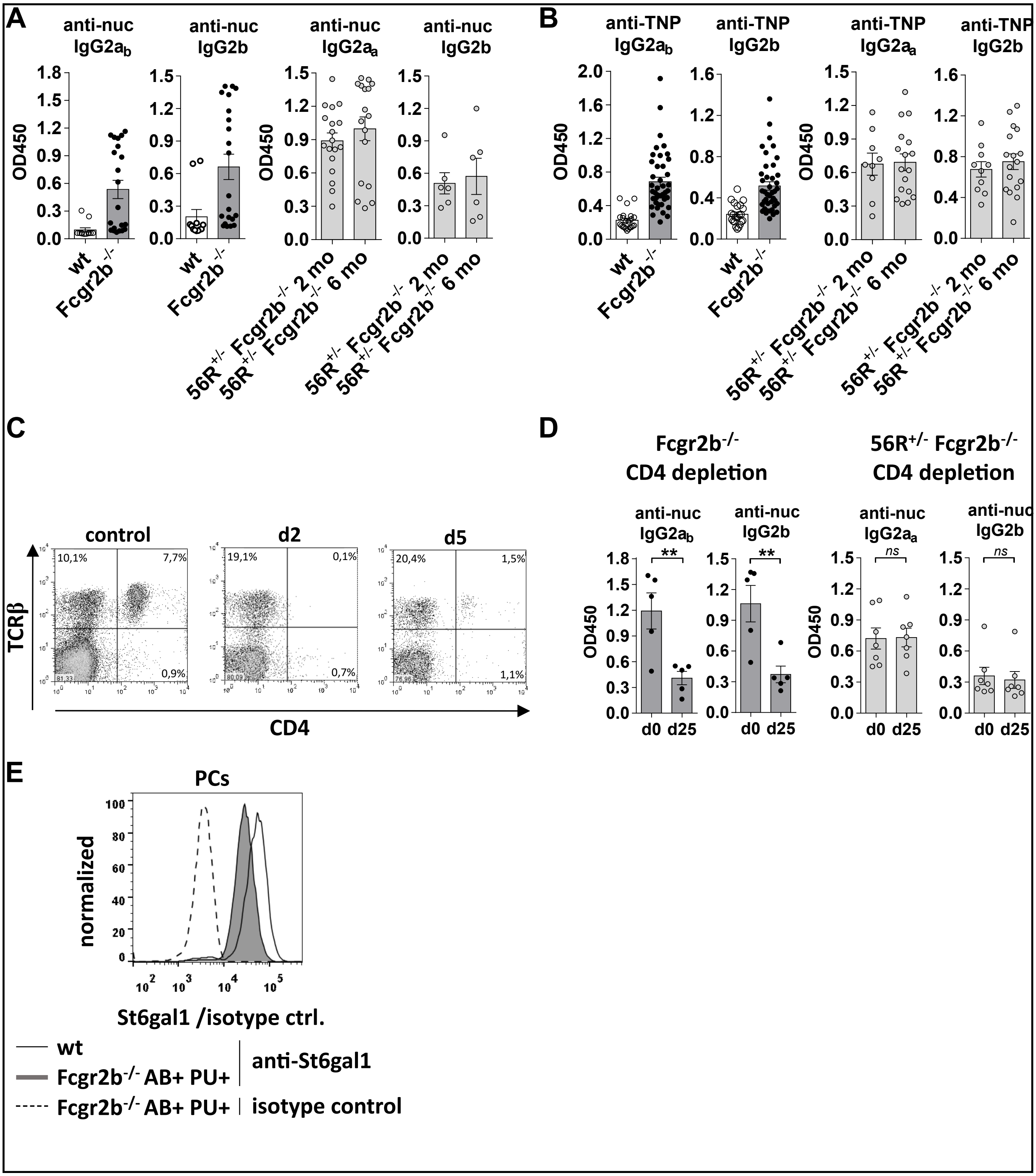
A
anti-nuc IgG2ab
anti-nuc IgG2b
anti-nuc IgG2aa
anti-nuc IgG2b
OD450
wt
Fcgr2b-/-
56R+/- Fcgr2b-/- 2 mo
56R+/- Fcgr2b-/- 6 mo
B
anti-TNP IgG2ab
anti-TNP IgG2b
anti-TNP IgG2aa
anti-TNP IgG2b
C
control
d2
d5
TCRβ
CD4
10,1%
7,7%
0,9%
19,1%
0,1%
0,7%
20,4%
1,5%
1,1%
D
Fcgr2b-/- CD4 depletion
56R+/- Fcgr2b-/- CD4 depletion
**
ns
d0
d25
E
PCs
normalized
St6gal1 /isotype ctrl.
wt
Fcgr2b-/- AB+ PU+
Fcgr2b-/- AB+ PU+
anti-St6gal1
isotype control

Fig. S2

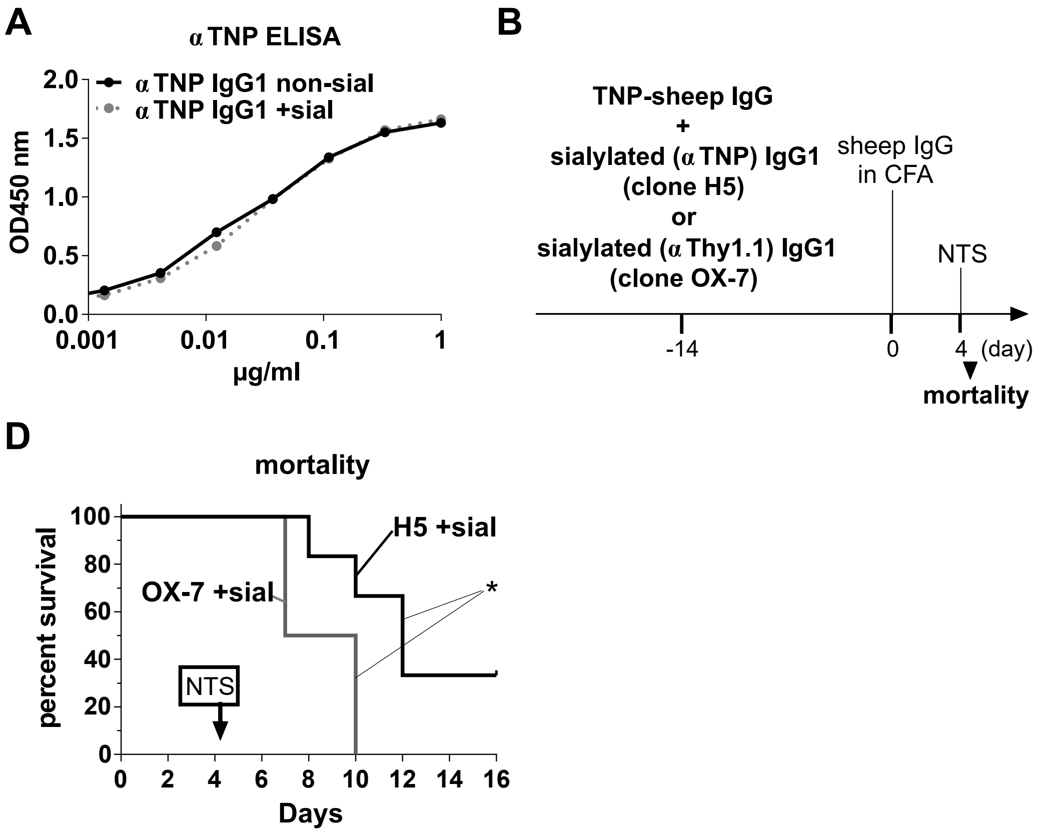
A
α TNP ELISA
α TNP IgG1 non-sial
α TNP IgG1 +sial
OD450 nm
μg/ml
B
TNP-sheep IgG
+
sialylated (α TNP) IgG1
(clone H5)
or
sialylated (α Thy1.1) IgG1
(clone OX-7)
sheep IgG
in CFA
NTS
-14
0
4
(day)
mortality
C
IgG1 sialylation
S2
S1
% of total glycans
OX-7 +sial
H5 +sial
D
mortality
percent survival
H5 +sial
OX-7 +sial
*
NTS
Days

Fig. S3

**A** **Col II-reactive (clone M2139) murine IgG1 heavy chain (MH208236):**

*Start* ↓ ... *AgeI* ↓

**Leader:** ATGGGATGGTCATGTATCATCCTTTTTCTAGTAGCAACTGCAA|CCGGTGTACATTCC

**VDJ:** <u>**CAGGTCCAGCTGCAGCAGTCTGGGGCTGAACTGGCAAGACCTG**</u>GGACCTCAGTGAAGATG
TCCTGCAAGGCTTCTGGCTACGCCTTTATTAGCTACTGGATGAACTGGGTAAAACAGAGGCCTG
GACAGGGTCTGGAATGGATTGGGGCTATTAATCCTAGCGATGGTTATACTGAGTACAATCAAA
AGTTCAAGGACAAGGCCATAATGACTGCAGACAGATCCTCCAGCACAGCCTACATGCAACTGA
GCAGCCTTACATCTGAGGACTCTGCACTCTATTACTGTGCAAGATATGGTGGATACTTTGACTA
CTGGGGCCAAGGCACCACTCTCACAGTCTCCTCA

*SalI* ↓

**IgG1 Fc:** GCG|TCGACGACACCCCCATCTGTCTATCCACTGGCCCCTGGATCTGCTGCCCAAACTAACTCCA
TGGTGACCCTGGGATGCCTGGTCAAGGGCTATTTCCCTGAGCCAGTGACAGTGACCTGGAACT
CTGGATCCCTGTCCAGCGGTGTGCACACCTTCCCAGCTGTCCTGCAGTCTGACCTCTACACTCT
GAGCAGCTCAGTGACTGTCCCCTCCAGCACCTGGCCCAGCCAGACCGTCACCTGCAACGTTGCC
CACCCGGCCAGCAGCACCAAGGTGGACAAGAAAATTGTGCCCAGGGATTGTGGTTGTAAGCCT
TGCATATGTACAGTCCCAGAAGTATCATCTGTCTTCATCTTCCCCCCAAAGCCCAAGGATGTGC
TCACCATTACTCTGACTCCTAAGGTCACGTGTGTTGTGGTAGACATCAGCAAGGATGATCCCGA
GGTCCAGTTCAGCTGGTTTGTAGATGATGTGGAGGTGCACACAGCTCAGACGAAACCCCGGGA
GGAGCAGATCAACAGCACTTTCCGTTCAGTCAGTGAACTTCCCATCATGCACCAGGACTGGCTC
AATGGCAAGGAGTTCAAATGCAGGGTCAACAGTGCAGCTTTCCCTGCCCCCATCGAGAAAACC
ATCTCCAAAACCAAAGGCAGACCGAAGGCTCCACAGGTGTACACCATTCCACCTCCCAAGGAG
CAGATGGCCAAGGATAAAGTCAGTCTGACCTGCATGATAACAAACTTCTTCCCTGAAGACATT
ACTGTGGAGTGGCAGTGGAATGGGCAGCCAGCGGAGAACTACAAGAACACTCAGCCCATCAT
GGACACAGATGGCTCTTACTTCGTCTACAGCAAGCTCAATGTGCAGAAGAGCAACTGGGAGGC
AGGAAATACTTTCACCTGCTCTGTGTTACATGAGGGCCTGCACAACCACCATACTGAGAAGAG
CCTCTCCCACTCTCCTGGTAAATGACGC|GTACG

↑ *Stop* ↑ *BsiWI*

**B** **Col II-reactive (clone M2139) murine kappa chain (MH208237):**

*Start* *AgeI*

**Leader:** **ATG**GGATGGTCATGTATCATCCTTTTTCTAGTAGCAACTGCAACCGGTGTACATTCA

**VJ:** **GACATTGTGCTCACCCAATCTCCAGCT**TCTTTGGCTGTGTCTCTAGGGCAGAGAGCCACCAT

CTCCTGCAGAGCCAGTGAAAGTGTTGAATATTTTGGCACGAGTTTAATGCAGTGGTACCAACA

GAAACCAGGACAGCCACCCAAACTCCTCATCTATGCTGCATCCAACGTAGAATCTGGGGTCCC

TGCCAGGTTTAGTGGCAGTGGGTCTGGGACAGACTTCAGCCTCAACATCCATCCTGTGGAGGA

GGATGATATTGCAATGTATTTCTGTCAGCAAAGTAGGGAGGTTCCGTACACGTTCGGAGGGGG

G**ACCAAGCTGGAAATAAAA**

*BsiWI*

**kappa:** CGTACGGATGCTGCACCAACTGTATCCATCTTCCCACCATCCAGTGAGCAGTTAACATCTGGA

GGTGCCTCAGTCGTGTGCTTCTTGAACAACTTCTACCCCAAAGACATCAATGTCAAGTGGAAGA

TTGATGGCAGTGAACGACAAAATGGCGTCCTGAACAGTTGGACTGATCAGGACAGCAAAGAC

AGCACCTACAGCATGAGCAGCACCCTCACGTTGACCAAGGACGAGTATGAACGACATAACAGC

TATACCTGTGAGGCCACTCACAAGACATCAACTTCACCCATTGTCAAGAGCTTCAACAGGAAT

GAGTGT**TGA**AAGCTT

*HindIII*

*Stop*

Fig. S4

## C Col II-reactive (clone CII 1-5) murine IgG1 heavy chain (MH208238):

*Start* *AgeI*

**Leader:** **ATG**GGATGGTCATGTATCATCCTTTTTCTAGTAGCAACTGCAA|CCGGTGTACATTCC

**VDJ:**
CAGGTGCAACTGCAGCAGTCAGGCGCTGAACTGGCAAAACCTGGGACCTCAGTGAAGATGTCC
TGCAAGGCTTCTGGCTACACCCTTATTAGTTACTGGATGAACTGGGTAAAACAGAGGCCTGGA
CAGGGTCTGGAATGGATTGGGGCTATTAATCCTAGCAATGGTTATACTGAGTACAATCAGAAG
TTCAAGGACAAGGCCATATTGACTGCAGACAAATCCTCCAGCACAGCCTACATGCAACTGAGC
AGCCTGACATCTGAGGACTCTGCAGTCTATTACTGTGCAAGAGAGGACTACGGTAGTACCCAC
TTTGACTACTGGGGCCAA**GGCACCACTCTCACAGTCTCCTCA**

*SalI*

**IgG1 Fc:**
GCG|TCGACGACACCCCCATCTGTCTATCCACTGGCCCCTGGATCTGCTGCCCAAACTAACTCCA
TGGTGACCCTGGGATGCCTGGTCAAGGGCTATTTCCCTGAGCCAGTGACAGTGACCTGGAACT
CTGGATCCCTGTCCAGCGGTGTGCACACCTTCCCAGCTGTCCTGCAGTCTGACCTCTACACTCT
GAGCAGCTCAGTGACTGTCCCCTCCAGCACCTGGCCCAGCCAGACCGTCACCTGCAACGTTGCC
CACCCGGCCAGCAGCACCAAGGTGGACAAGAAAATTGTGCCCAGGGATTGTGGTTGTAAGCCT
TGCATATGTACAGTCCCAGAAGTATCATCTGTCTTCATCTTCCCCCCAAAGCCCAAGGATGTGC
TCACCATTACTCTGACTCCTAAGGTCACGTGTGTTGTGGTAGACATCAGCAAGGATGATCCCGA
GGTCCAGTTCAGCTGGTTTGTAGATGATGTGGAGGTGCACACAGCTCAGACGAAACCCCGGGA
GGAGCAGATCAACAGCACTTTCCGTTCAGTCAGTGAACTTCCCATCATGCACCAGGACTGGCTC
AATGGCAAGGAGTTCAAATGCAGGGTCAACAGTGCAGCTTTCCCTGCCCCCATCGAGAAAACC
ATCTCCAAAACCAAAGGCAGACCGAAGGCTCCACAGGTGTACACCATTCCACCTCCCAAGGAG
CAGATGGCCAAGGATAAAGTCAGTCTGACCTGCATGATAACAAACTTCTTCCCTGAAGACATT
ACTGTGGAGTGGCAGTGGAATGGGCAGCCAGCGGAGAACTACAAGAACACTCAGCCCATCAT
GGACACAGATGGCTCTTACTTCGTCTACAGCAAGCTCAATGTGCAGAAGAGCAACTGGGAGGC
AGGAAATACTTTCACCTGCTCTGTGTTACATGAGGGCCTGCACAACCACCATACTGAGAAGAG
CCTCTCCCACTCTCCTGGTAAA**TGA**CGC|GTACG

*Stop* *BsiWI*

Fig. S4 ↑

**D** **Col II-reactive (clone CII 1-5) murine kappa chain (MH208239):**

*Start* ↓ *AgeI* ↓

**Leader:** ATGGGATGGTCATGTATCATCCTTTTTCTAGTAGCAACTGCAA|CCGGTGTACATTCA

**VJ:** GACATC**C**AG**A**T**G**ACTCAGTCTCCA**G**CCTCCCTATCTGCATCTGTGGGAGAAACTGTCACCATCA

CATGTCGAGCAAGTGAGAATATTTACAGTTATTTAGCATGGTATCAGCAGAAACAGGGAAAAT

CTCCTCAGCTCCTGGTCTATAATGCAAAAACCTTAGCAGAAGGTGTGCCATCAAGGTTCAGTGG

CAGTGGATCAGGCACACAGTTTTCTCTGAAGATCAACAGCCTGCAGCCTGAAGATTTTGGGAG

TTATTACTGTCAACATCATTATGGTACTCCTCGGACGTTCGGTGGAGGGACC**AAGCTGGAAAT**

**CAAA**

*BsiWI* ↓

**kappa:** C|GTACGGATGCTGCACCAACTGTATCCATCTTCCCACCATCCAGTGAGCAGTTAACATCTGGA

GGTGCCTCAGTCGTGTGCTTCTTGAACAACTTCTACCCCAAAGACATCAATGTCAAGTGGAAGA

TTGATGGCAGTGAACGACAAAATGGCGTCCTGAACAGTTGGACTGATCAGGACAGCAAAGAC

AGCACCTACAGCATGAGCAGCACCCTCACGTTGACCAAGGACGAGTATGAACGACATAACAGC

TATACCTGTGAGGCCACTCACAAGACATCAACTTCACCCATTGTCAAGAGCTTCAACAGGAAT

GAGTGTTTGAA|AGCTT

↑ *Stop* ↑ *HindIII*

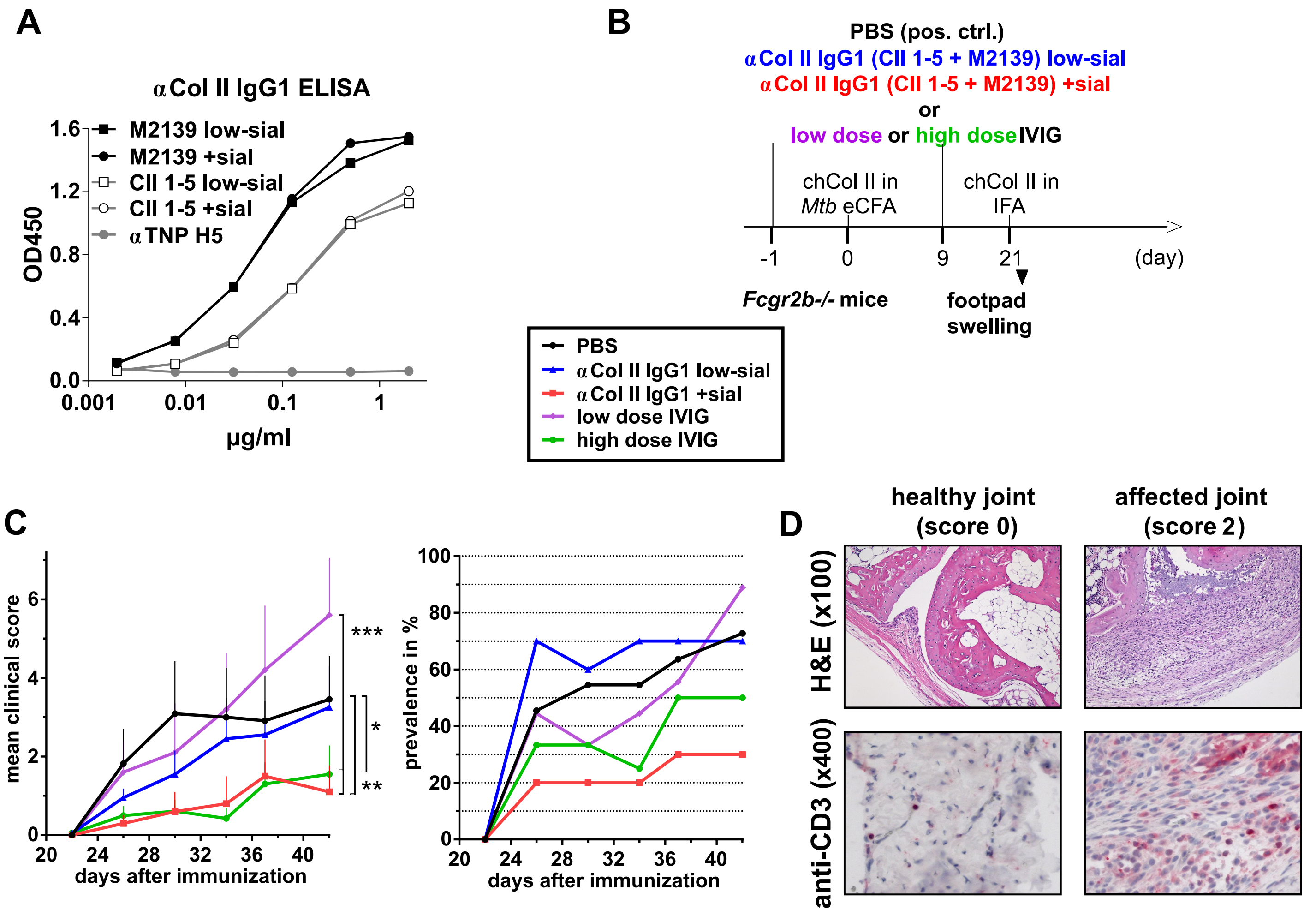

A
αCol II IgG1 ELISA
M2139 low-sial
M2139 +sial
CII 1-5 low-sial
CII 1-5 +sial
αTNP H5
OD450
µg/ml
B
PBS (pos. ctrl.)
αCol II IgG1 (CII 1-5 + M2139) low-sial
αCol II IgG1 (CII 1-5 + M2139) +sial
or
low dose or high dose IVIG
chCol II in Mtb eCFA
chCol II in IFA
(day)
Fcgr2b-/- mice
footpad swelling
PBS
αCol II IgG1 low-sial
αCol II IgG1 +sial
low dose IVIG
high dose IVIG
C
mean clinical score
days after immunization
prevalence in %
days after immunization
D
healthy joint (score 0)
affected joint (score 2)
H&E (x100)
anti-CD3 (x400)


Fig. S5

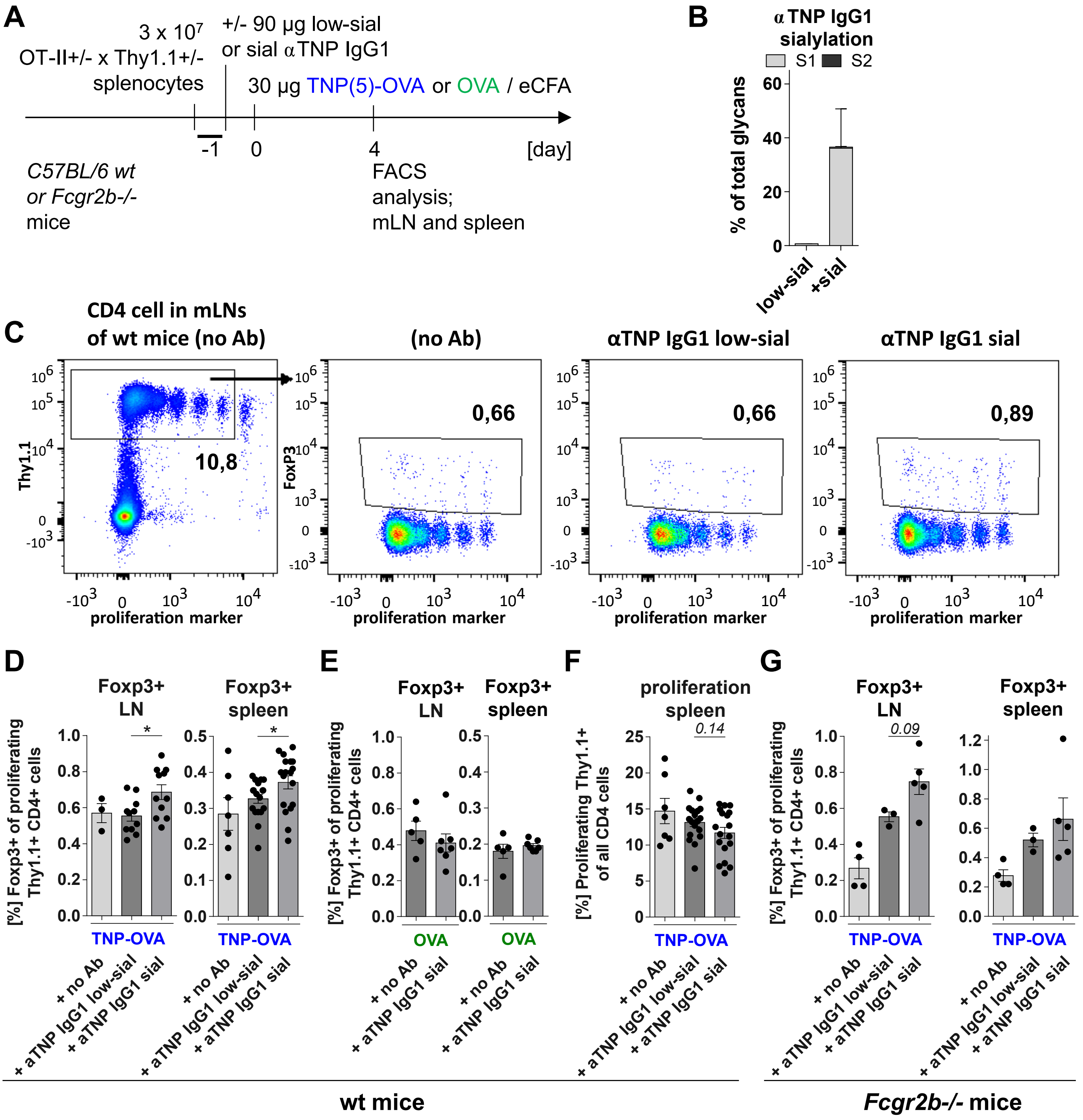

A
3 x 10^7 OT-II+/- x Thy1.1+/- splenocytes
+/- 90 µg low-sial or sial αTNP IgG1
30 µg TNP(5)-OVA or OVA / eCFA
-1
0
4
[day]
C57BL/6 wt or Fcgr2b-/- mice
FACS analysis; mLN and spleen
B
α TNP IgG1 sialylation
S1
S2
% of total glycans
low-sial
+sial
C
CD4 cell in mLNs of wt mice (no Ab)
(no Ab)
αTNP IgG1 low-sial
αTNP IgG1 sial
Thy1.1
FoxP3
10,8
0,66
0,66
0,89
proliferation marker
D
Foxp3+ LN
Foxp3+ spleen
[%] Foxp3+ of proliferating Thy1.1+ CD4+ cells
TNP-OVA
+ no Ab
+ aTNP IgG1 low-sial
+ aTNP IgG1 sial
E
Foxp3+ LN
Foxp3+ spleen
OVA
+ no Ab
+ aTNP IgG1 sial
F
proliferation spleen
0.14
[%] Proliferating Thy1.1+ of all CD4 cells
G
Foxp3+ LN
0.09
Foxp3+ spleen
wt mice
Fcgr2b-/- mice


Fig. S6
